# Monodisperse oligo(ε-caprolactones) with terpenes and alkyl end-groups: synthesis, isolation, characterization, and antibacterial activity†

**DOI:** 10.1039/d4ra08104h

**Published:** 2025-01-02

**Authors:** María Guadalupe Ortiz-Aldaco, Miriam Estévez, Beatriz Liliana España-Sánchez, José Bonilla-Cruz, Eloy Rodríguez-deLeón, José E. Báez

**Affiliations:** a Department of Chemistry, University of Guanajuato (UG) Noria Alta S/N 36050 Guanajuato Gto Mexico jebaez@ugto.mx; b Centro de Fisica Aplicada y Tecnología Avanzada (CFATA), UNAM Juriquilla Qro. Mexico; c Centro de Investigación y Desarrollo Tecnológico en Electroquímica (CIDETEQ) Pedro Escobedo Qro. Mexico; d Centro de Investigación en Materiales Avanzados S.C. (CIMAV), Unidad Monterrey Mexico; e Universidad Autónoma de Querétaro (UAQ) Querétaro Qro. Mexico

## Abstract

Linear aliphatic oligoesters derived from ε-caprolactone (CL) were synthesized by ring-opening polymerization (ROP) using terpene alcohols that have antibacterial activity as initiators (nerol, geraniol, β-citronellol and farnesol). Ammonium decamolybdate (NH_4_)_8_[Mo_10_O_34_] was used as a catalyst. From previous oligoesters, monodisperse species of monomers, dimers, and trimers were isolated by flash column chromatography (FCC). Poly(ε-caprolactone) (PCL) oligoesters [oligo(CLs)] and monodisperse oligomeric species were characterized by different analytical techniques, such as nuclear magnetic resonance (NMR) spectroscopy, electrospray ionization quadrupole time-of-flight mass spectrometry (ESI/MS-QTOF), and Fourier-transform infrared (FTIR) spectroscopy to determine the chemical nature of the samples. The thermal properties were analyzed by differential scanning calorimetry (DSC), which showed significant differences between the olefin and alkyl terminal groups. The end-groups affected crystalline domains according to the crystallization temperatures (*T*_c_), melting temperatures (*T*_m_), and glass transition temperature (*T*_g_) of the oligo(CLs) and monodisperse oligomeric species. In addition, the results of thermogravimetric analysis (TGA) suggest that the thermal degradation in the case of the monomer and dimer species with olefin terminal groups is similar compared to that with the alkyl terminal group. Due to the antimicrobial properties of olefinic initiators, microbiological tests were carried out on the monodisperse oligomeric species through studies of the minimum inhibitory concentration (MIC), minimum bactericidal concentration (MBC), and antibiograms. This is the first time in the literature that monodisperse oligomers derived from PCL functionalized with terpenes and alkyl end-groups were tested in terms of their antibacterial properties. The results indicated that these monodisperse species could lead to new antibiotic compounds with potential applications.

## Abstract 2

A systematic study of monodisperse oligoesters derived from ε-caprolactone with terpene and alkyl terminal groups showing antibacterial activity.

## Graphical abstract

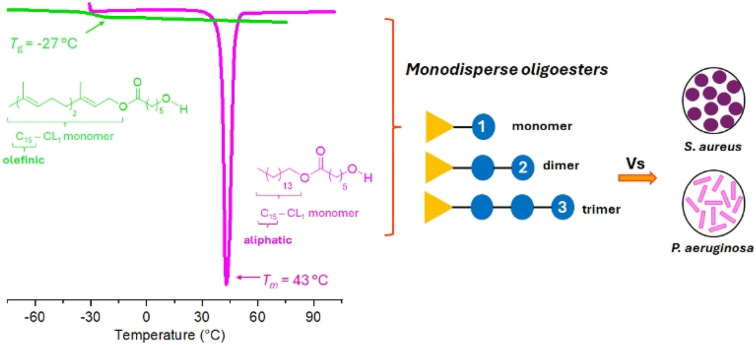

## 1. Introduction

Biodegradable polyesters such as poly(ε-caprolactone) (PCL) are of great interest due to their biocompatibility and biodegradability.^1^ PCL can be prepared by ring-opening polymerization (ROP) of ε-caprolactone (CL) using an alcohol (R-OH)^2–4^ [or diol (HO-R-OH)]^5–9^ as an initiator. The alkyl substituent becomes a terminal group and leads to α-hydroxyl-ω-alkyl-PCL (HO-PCL-R). This phenomenon is also applied to other monomers such as δ-valerolactone (δ-VL),^2^ glycolide (GA),^4^ and l-lactide (l-LA).^4,10,11^ A homopolymer of CL is often used in biomedicine due to its hydrophobic character when used in biodegradable stents^12^ in soft tissues and drug delivery.^13^ There is growing academic and industrial interest in biodegradable antimicrobial oligomeric polyesters, but few studies have been carried out on oligomers based on PCL.^14^ However, in recent years, efforts have been made to separate monodisperse species, including PCL.

Stepwise synthesis methods (linear and exponential growth) have been very successful in the production of these oligomers, but these methods result in reduced separation with higher-molecular-weight oligomers and decreased statistical yield of any one oligomer length at a higher degree of polymerization (DP). Furthermore, the multi-step nature is inefficient for preparing large libraries of discrete oligomers and can be challenging. For such purposes, separation methods are used to isolate individual oligomers from dispersed materials, such as flash column chromatography (FCC).^2,15–19^

Terpenes are the most abundant family of organic natural products in nature and are the main constituents of essential oils. They have a skeleton that is formed by isoprene and oxygen units, and they are produced and secreted by specialized plant tissues.^20^ Terpenes may present various organic functional groups, such as alcohols, ketones, ethers, esters, and aldehydes,^21^ and they have biological activities that are related to their functional groups, arrangements, and structures.^22^ These natural hydrocarbons are made up of five carbon isoprene units with different configurations, various degrees of unsaturation, oxidation, functional groups, and rings.^23,24^ For example, nerol and geraniol are aliphatic monoterpene structures (C_10_H_18_O) produced by the combination of two isoprene units (C_5_H_8_) that have a functional alcohol group in their organic composition, while farnesol (C_15_H_26_O) is a sesquiterpene alcohol with three isoprene units.

Nerol is extracted from Damask rose (*Rosa damascena*), *Lavandula stoechas*, *Lavandula multifida*, and lemongrass (*Cymbopogon citratus*).^25^ β-Citronellol is a part several volatile oils in plants of the genus *Cymbopogon* (such as *Cymbopogon nardus*).^26–28^ Geraniol is a part of various volatile oils and a principle product of *Cymbopogon martinii* (66.2–76.9%),^29^*Pelargonium graveolens* (21.08%),^30^*R. damascena* (18.7–21.2%), *Rosa centifolia* (7.4–11.3%),^31^ and *Cymbopogon nardus* (22.77%).^32^ This phytoconstituent has been reported to have biological and pharmacological properties, such as antibacterial activity.^33,34^ The mechanism of this activity is based on its lipophilic character and is explained by the ability to adhere to cell membrane lipids of the microorganism. This allows it to interact with the organism's components, make it more permeable, bind essential intracellular sites, and thus destroy its structures.^33,35^ These terpene compounds exhibit good antimicrobial activity against *Staphylococcus aureus* and *Escherichia coli*.^23,36–39^

One of the main drawbacks of PCL is its lack of functional groups with antimicrobial activity, which is why it has been possible to synthesize biodegradable antimicrobial PCL.^40–54^ To improve the antibacterial behavior of PCL, natural compounds have been incorporated as additives, such as resveratrol,^55^ polyhexamethylene guanidine derivatives,^56^ and essential oils such as cinnamaldehyde and allyl isothiocyanate.^57^ Jummes *et al.* synthesized PCL nanoparticles that entrapped palmarosa essential oil and its majoritarian compound, geraniol, resulting in antimicrobial activity against *E. coli* and *S. aureus*.^58^

PCL is a biodegradable polyester with oligomers that can be recognized as a carbon source or degraded by enzymes of different microorganisms. Thus, we have been examining the fusion between two different types of chemical species: biodegradable polyesters, such as PCL, and organic molecules with biological properties, such as terpenes. Some of these terpenes have an alcohol functional group such as nerol, geraniol, β-citronellol, or farnesol and can act as initiators in the ROP of CL.

We examined what roles terpenic molecules have as a terminal group in a monodisperse oligoester derived from PCL, as well as the effect of the end-group on the physical properties of the monodisperse oligomer. We also examined whether a monodisperse oligomer with a terpenic end-group can retain the biological properties of its terpene precursor, as well as the ability of such oligomers to act as a “trojan horse” against bacteria. We report the synthesis, isolation, characterization, and antimicrobial evaluation of monodisperse oligomeric species derived from PCL and functionalized terpene alcohols that have antibacterial activity (nerol, geraniol, β-citronellol, and farnesol).

The terpenes were inserted as end-groups by ROP of the CL. Monodisperse oligomeric species from monomer to trimer were isolated by FCC and analyzed by a range of characterization techniques to examine the chemical nature and the effect of DP on the physical properties of the oligomer. Additionally, we compared the differences between the terpene farnesol (C_15_) and aliphatic 1-pentadecanol (C_15_) as a terminal group of monodisperse PCL. Microbiological tests were also carried out using Gram-positive bacteria *S. aureus* and Gram-negative *Pseudomonas aeruginosa*.

## 2. Experimental

### 2.1. Materials

All reagents, ε-caprolactone (CL), nerol, geraniol, β-citronellol, farnesol and 1-pentadecanol, ammonium decamolybdate [(NH_4_)_8_(Mo_10_O_34_)] and deuterated chloroform (CDCl_3_) were purchased from Sigma Aldrich Co. (St Louis, MO, USA) and used as received. Thin-layer chromatography (TLC) was performed on percolated silica gel plates and using a Seebach staining reagent. Flash column chromatography (FCC) was conducted using 230–400 mesh silica gel. Toluene and ethyl acetate were used as the mobile phase during FCC. For the antibacterial assays, strains of *Staphylococcus aureus* #6538 and *Pseudomonas aeruginosa* #13388 from American Type Culture Collection (ATCC) were growth in Mueller Hinton broth (BD Bioxon).

### 2.2. Instruments

Nuclear magnetic resonance (NMR) spectroscopy. Solution state ^1^H and ^13^C spectra were recorded at room temperature or above on a 500 MHz Bruker Avance III HD instrument, using CDCl_3_ as a solvent. Chemical shifts are reported as *δ* in parts per million (ppm) and referenced to the chemical shift of the residual solvent (^13^C at *δ* 77.16, and ^1^H at *δ* 7.26, for CDCl_3_). FT-IR spectra were recorded on a PerkinElmer Spectrum 100 FTIR spectrophotometer with attenuated total reflectance spectroscopy (ATR) accessory. Size exclusion chromatography (SEC). All polyester samples were dissolved in THF (5 mg/5 mL) heating at 37 °C for one hour and filtered with an 0.45 μm Acrodisc®. The SEC instrument (Agilent) was equipped with a refractive index detector. Measurements were determined using a single column PLgel 5 μm Mixed-D (Agilent) at a flow rate of 1.0 mL min^−1^ with HPLC-grade THF. Polystyrene standards (Polymer Laboratories) were used for calibration. Thermograms were performed in two different instruments, the first a Differential Scanning Calorimetry (DSC) Q200 V24.11 Build 124 instrument (an intracooler at −30 °C), the second similar to the first one but with an intracooler at −90 °C. For the oligomers: three scans were obtained with two heating scans (25 to 170 °C and −30 to 170 °C) and one cooling scan (170 to −30 °C) between them. For the monodisperses species: three scans were obtained with two heating scans (25 to 80 °C and −85 to 75 °C) and one cooling scan (80 to −85 °C) between them. The rate of heating/cooling was 10 °C min^−1^ and was performed under a nitrogen purge. The glass transition temperature (*T*_g_) is given as an inflection point, and the melting points (*T*_m_) are given as the minimum of the endothermic transition, and the data presented are taken from the second heating scan. Electrospray ionization quadrupole mass (ESI/MS-QTOF) spectroscopy in positive ionization mode using ESI-Q-TOF-MS (Waters – Synapt G1) equipped. The carrier gas was nitrogen with a flow rate of 1.5 mL min^−1^. The sample volume injected was 250 μL. The temperature of the injector was held at 120 °C and the transfer line at 300 °C. The mass spectrometer was operated at 2 V ionization energy, the spectra were recorded in scan mode in the range 50–1200 *m*/*z*. The spectra obtained were visualized in the program Mass Lynx V4.1 (Waters), and the ions produced are inspected. Gas Chromatography-Mass Spectrometry (GC-MS), using an Agilent model 6850 gas chromatograph coupled to a 5973N mass spectrometer with a single quadrupole detector (Agilent technologies, Palo Alto, CA, USA). Chromatographic separation was performed on a HP-5 capillary column Agilent (30 m × 20 mm × 0.25 μm). The carrier gas was helium with a flow rate of 1 mL min^−1^. The sample volume injected was 1 μL and the split ratio 1 : 50. The oven temperature started at 50 °C for 1 minute, then increased to 250 °C at 10 °C min^−1^. The temperature of the injector was held at 150 °C and the transfer line at 250 °C. The mass spectrometer was operated at 70 eV ionization energy, the spectra were recorded in scan mode in the range 100–280 *m*/*z*. Polarized optical microscopy (POM). POM micrographs were obtained using a Nikon ECLIPSE E200 optical microscope; photographs were taken using an iPhone 13 mini. All samples were collected with a magnification of 40×. Thermal and decomposition characteristics of the monodisperses species (C_15_F-CL_1_, and C_15_1P-CL_1_, Fig. 12; C_15_F-CL_2_, and C_15_1P-CL_2_, Fig. S27†) were determined by thermogravimetric analysis (TGA), conducted on a STAR^e^ SW 13.00 TGA/DSC 2 of METTLER TOLEDO, in the temperature range of 35–595 °C with a heating rate of 10 °C min^−1^ under a flow of nitrogen at 40 mL min^−1^.

### 2.3. Synthesis of oligo(CLs) (DP_theo_ = 1)

Polymerization was performed in a previously dried 25 mL round-bottom flask. ε-Caprolactone (CL) (10 mmol, 1.1414 g), ammonium heptamolybdate tetrahydrate (NH_4_)_6_[Mo_7_O_24_]·4H_2_O (Hep, 1.21 × 10^−3^ mmol, 1.5 mg), and an initiator were charged and heated to aluminum block at 150 °C for 1.5 hour. By thermal decomposition *in situ* of ammonium heptamolybdate (NH_4_)_6_[Mo_7_O_24_], ammonium decamolybdate (NH_4_)_8_[Mo_10_O_34_] was obtained in the solid-state.^59^ The oligo(CLs) obtained and analyzed were isolated without purification (yield = 96–99%). The different initiators: geraniol (C_10_) (10 mmol, 1.54 g), nerol (C_10_) (10 mmol, 1.54 g), β-citronellol (C_10_) (10 mmol, 1.56 g), farnesol (C_15_) (10 mmol, 2.22 g), and 1-pentadecanol (10 mmol, 2.28 g) [CL/initiator = 1]. Isolation of oligomers by flash column chromatography: 600 mg of oligo(CL) (DP_theo_ = 1) was dissolved in the minimum volume of toluene (5 mL) and added to a silica gel column with toluene used as mobile phase. Gradually, the fraction of toluene/ethyl acetate increased to 90/10, 80/20 and 75/25. All the fractions were analyzed by thin-layer chromatography (TLC mobile phase: toluene/ethyl acetate = 80/20) and visualizing the spots using the Seebach staining reagent. The fractions were collected in test tubes, the solvent was evaporated by a rotary evaporator, and the resulting oil (or solid) was dried overnight under vacuum. Fractions: monomer C_10_C-CL_1_ [226.0 mg, wt% = 34.27%, mol% = 11.83% (mol% C_10_OH = 41.20%, mol% CL = 58.8%)], dimer C_10_C-CL_2_ [181.2 mg, wt% = 27.48%, mol% = 20.48% (mol% C_10_OH = 29.68%, mol% CL = 70.32%)], and trimer C_10_C-CL_3_ [71.1 mg, wt% = 10.78%, mol% = 67.69% (mol% C_10_OH = 22.89%, mol% CL = 77.11%)]. NMR data at room temperature: ^1^H NMR (500 MHz, CDCl_3_, ppm). C_10_C-CL_1_ (Fig. S9†): *δ* 5.08 (t, 1H, [*k*, =CH–], C_10_), 4.10 (quintet, 2H, [*f*, –CH_2_–O–], C_10_), 3.65 (t, 2H, [*a*, –CH_2_–OH], CL_1_), 2.31 (t, 2H, [*d*, –CH_2_–CO–], CL_1_), 1.98 (quintet, 2H, [*j*, –CH_2_–CH=], C_10_), 1.68 (quintet, 3H, [*l*, CH_3_–], C_10_), 1.65 (quintet, 2H, [*i*, –CH_2_–CH_2_–], C_10_), 1.64 (quintet, 2H, [*b*, –CH_2_–CH_2_–], CL), 1.60 (quintet, 3H, [*n*, CH_3_–], C_10_), 1.40 (quintet, 2H, [*g*, –CH_2_–(CH)–], C_10_), 1.33 (quintet, 2H, [*c*, –CH_2_–CH_2_–], CL_1_), 1.19 (quintet, 1H, [*h*, –CH–(CH_3_)–CH_2_–], C_10_), 0.91 (quintet, 1H, [*m*, CH_3_–(CH)–], C_10_). FT-IR: C_10_C-CL_1_ (Fig. S19†).

### 2.4. Antibacterial assays

The quantitative *in vitro* antibacterial activity of monodisperse oligomeric species was performed by broth microdilution, according to the CLSI M 07 method (methods for dilution antimicrobial susceptibility tests for bacteria that grow aerobically) in contact with inoculum Gram-positive *S. aureus* and Gram-negative *P. aeruginosa*. For this purpose, different amount of each sample (4, 8, 16, 32, 64, 128, and 256 μg mL^−1^) were suspended in dimethyl sulfoxide (DMSO) to improve the oligomer dispersion. Ciprofloxacin was used as reference for antimicrobial agent. Inoculum of each microorganism were growth in MH broth for 16 h/37 °C and adjusted to 1 × 10^5^ colony unit forming (CFU mL^−1^). An equal volume (500 μL) of bacteria/oligomer suspension was placed in a sterile Eppendorf tube and stirred at 37 °C for 24 h. After interaction, an aliquot (50 μL) was plated in MH agar and the minimum inhibitory concentration (MIC) and the bactericidal concentration (CMB) was calculated, compared with a positive control (bacterial growth).

## 3. Results and discussion

### 3.1. Oligomers derived from oligo(ε-caprolactone) with olefinic and aliphatic end-groups

**Scheme 1:**
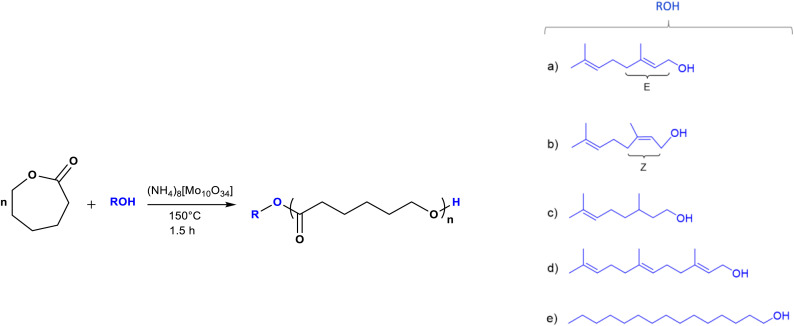
Synthesis of oligo(ε-caprolactones) [oligo(CLs)] catalyzed by ammonium decamolybdate [(NH_4_)_8_(Mo_10_O_34_)] using different types of initiators (R-OH): (a) geraniol (C_10_), (b) nerol (C_10_), (c) β-citronellol (C_10_), (d) farnesol (C_15_), and (e) 1-pentadecanol (C_15_).

Oligomers were synthesized by ROP of CL using ammonium decamolybdate (NH_4_)_8_[Mo_10_O_34_] as a catalyst and alcohols (ROH) as initiators: prenol, nerol, geraniol, β-citronellol, farnesol, and 1-pentadecanol (Scheme 1). To examine the effect of the terminal group on the properties of the oligo(CLs), the feed molar ratio of CL to ROH was set to 1. The main idea was to obtain a blend of different types of oligomers such as monomers, dimers, and trimers after the polymerization reaction.

Table 1 shows the results obtained for the oligo(CLs) synthesized after a reaction time of 1.5 h at 150 °C. These oligo(CLs) were successfully obtained with high conversion (from 96 to 99%). The experimental values of number-average molecular weight (*M*_n_) were obtained by nuclear magnetic resonance (NMR) spectroscopy and size exclusion chromatography (SEC). The *M*_n_(NMR) values were similar to those of *M*_n_(calcd). On the other hand, *M*_n_(SEC) was between 600 and 800 g mol^−1^ and was higher than *M*_n_(calcd). The *M*_n_(NMR)/*M*_n_(SEC) ratio was between 0.3 and 0.5.

**Table 1:** Oligoesters derived from ε-caprolactone (CL) [oligo(CLs)] using different types of aliphatic alcohols as initiators with a feed degree of polymerization (DP) of 1

No.	Sample	Initiator	MW^a^	*M*_n_ b (NMR)	*M*_n_ c (SEC)	DP^b^ (NMR)	*Đ*_M_ c ^,^ d	Conv.^b^ (%)	*R* e (%)	Ratio^f^	*T*_c_ g (°C)	*T*_m_ g (°C)	Δ*H*_m_^g^ (J g^−1^)
1	C_10_G-PCL	Geraniol	268	275	809	1.06	1.10	97	56	0.34	—	—	—
2	C_10_N-PCL	Nerol	268	268	688	1.0	1.08	98	57	0.39	−48	−31, −5	19
3	C_10_C-PCL	β-Citronellol	270	272	756	1.02	1.09	99	57	0.36	—	—	—
4	C_15_F-PCL	Farnesol	336	338	665	1.02	1.15	99	65	0.50	—	—	—
5	C_15_1P-PCL	1-Pentadecanol	342	342	—	1.0	—	96	66	—	—	8, 16, 27, 31	141

a Theoretical value (g mol^−1^).

b Determined by ^1^H NMR in CDCl_3_.

c Determined by size-exclusion chromatography (SEC) analysis.

d *Đ*_M_: dispersity.

e Obtained from the equation *R* (%) = (MW_initiator_/*M*_n_(NMR)) × 100, *R* = olefin or alkyl. Where MW_initiator_ is the molecular weight of initiator or alkyl diol (HOROH).

f *M*_n_(NMR)/*M*_n_(SEC) ratio.

g Obtained by DSC analysis.

In previous studies, *M*_n_(SEC) was found to overestimate the real *M*_n_ value,^59–61^ and *M*_n_(NMR) is usually more accurate for oligomers than *M*_n_(SEC). This effect is attributed to the differences in the hydrodynamic radius between polystyrene standards and PCL samples. The experimental DP detected by NMR showed similar numbers to the feed molar values of CL/ROH, which indicated the control of DP. All oligo(CLs) exhibited a high end-group values (wt% = 56–66%).

**Fig. 1:**
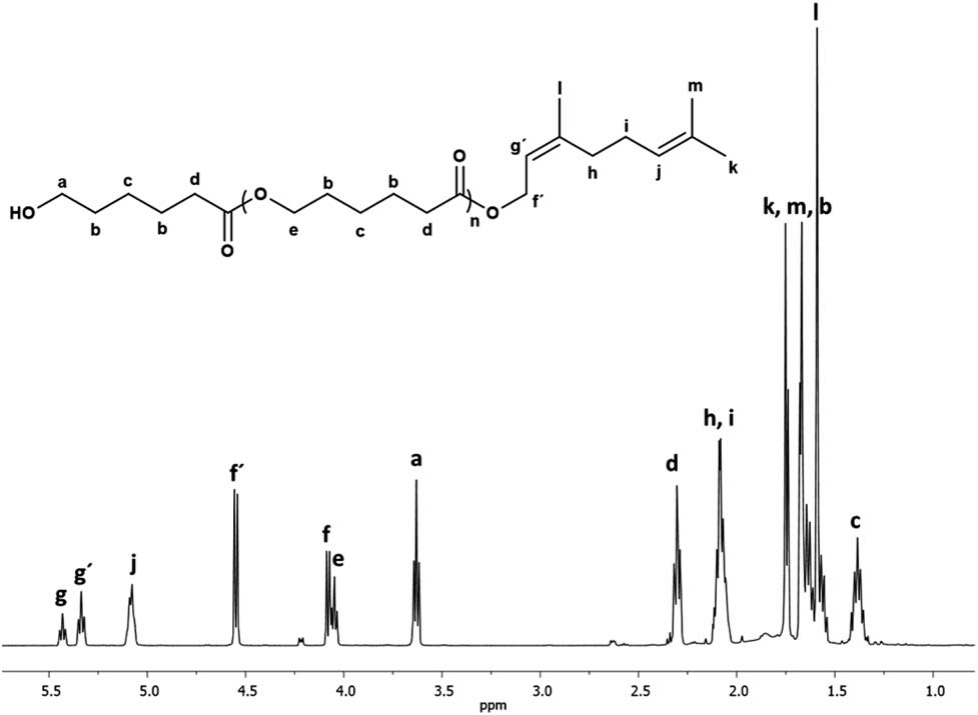
^1^H NMR (500 MHz) spectrum of oligo(CL) synthetized using nerol as initiator (C_10_N-PCL, Table 1) in CDCl_3_ at 40 °C. Signal f[methylene, HO–CH_2_–CH=C] and g[methine, HO–CH_2_–CH=C] are from unreacted nerol.

Fig. 1 shows the ^1^H NMR spectrum of the oligo(CL) synthetized using nerol as an initiator (C_10_N-PCL). The spectrum showed characteristic peaks for methylene attached to hydroxyl [*a*, –CH_2_OH, *δ* 3.63], and methyl end groups [*k*, –CH_3_, *δ* 1.75]. The repetitive units are attributed to the methylenes of the main chain of the polymer [*e*, –CH_2_–O−, *δ* 4.05 and *d*, –CH_2_–(C=O)–O−, *δ* 2.30], and the vinyl groups of the nerol [*g*′, *j*, –CH=C–, *δ* 5.34, 5.08] were clearly visible. Furthermore, signals corresponding to unreacted nerol were observed ([*f*, –CH_2_–, *δ* 4.08] and [*g*, –CH=C–, *δ* 5.43]). There was no evidence of the oxidation of olefinic groups (epoxy or secondary alcohol).

**Fig. 2:**
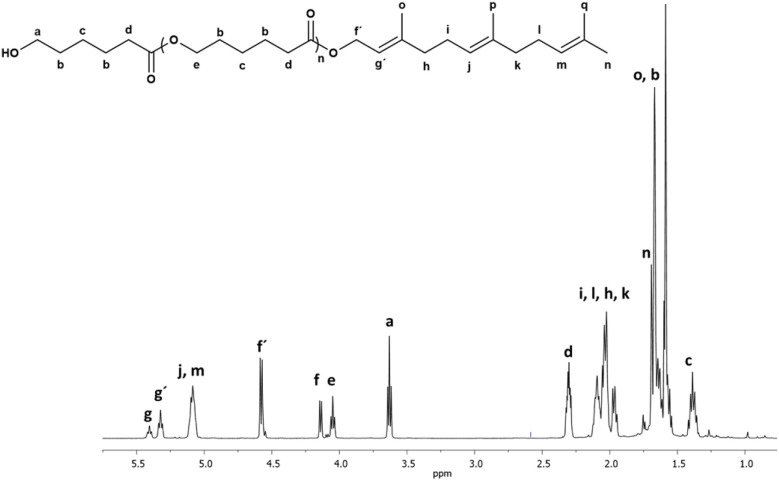
^1^H NMR (500 MHz) spectrum of oligo(CL) synthetized using farnesol as initiator (C_15_F-PCL, Table 1) in CDCl_3_ at 40 °C. Signal f[methylene, HO–CH_2_–CH=C] and g[methine, HO–CH_2_–CH=C] are from unreacted nerol.

Another oligomer obtained with farnesol as the initiator showed the same pattern of peaks for the PCL except for additional signals assigned to the longer olefin end-group (Fig. 2).

Thermal properties such as the glass transition temperature (*T*_g_), crystallization temperature (*T*_c_), and melting temperature (*T*_m_) were studied by differential scanning calorimetry (DSC) to examine the effect of olefin and alkyl groups on the oligo(CLs) (Table 1). The oligo(CLs) produced using geraniol, β-citronellol, and farnesol as initiators did not show any transition in the range of −85 to 75 °C, which suggests that the samples were amorphous. This effect is due to branched methyl and vinyl carbons that favored steric hindrance and rigidity, inducing an amorphous domain in the oligomer chains.

In the case of oligo(CL) with the olefin nerol as the initiator (C_10_N-PCL, *T*_c_ = −48, and *T*_m_ = −31, −5 °C), an unusual result was observed in comparison to the rest of the oligo(CLs) with olefinic terminal groups.

Nerol and geraniol are geometric isomers with *Z* and *E* configurations, respectively. It is likely that the branched methyl attached to the alcohol in the nerol is sterically less exposed in comparison to geraniol. Consequently, when nerol becomes a terminal group in the oligo(CL), it results in less steric hindrance of the blend of different chains of oligo(CL), which leads to a semicrystalline domain at low temperature (*T*_m_ = −31, −5 °C).

In contrast, an oligo(CL) with an alkyl terminal group (initiated with 1-pentadecanol, C_15_1P-PCL), there was an intense endothermic transition of *T*_m_ (8, 16, 27, 31 °C). Comparison between C_15_1P-PCL (alkyl end-group) and C_15_F-PCL (olefin end-group) showed different environments in terms of their morphologies, which were semicrystalline and mainly amorphous, respectively. It is evident that an alkyl terminal group such as pentadecyl (C_15_, C_15_1P-PCL) with *anti* conformation favored the crystallization of the oligo(CL). On the other hand, the olefinic end-group (C_15_, C_15_F-PCL) with sp^2^ carbons and branched methyl promotes amorphous domains.

### 3.2. Monodisperse species derived from oligo(ε-caprolactone) [oligo(CLs)]

The main purpose of the synthesis in this study is the possibility that the species examined could be the precursors of monodisperses oligomers (monomers, dimers, and trimers). The monodisperse species were gradually isolated from the crude reaction using thin layer chromatography (TLC). For this method, a mixture of toluene/ethyl acetate solvents (Experimental section) was effective in the separation of spots, and then flash column chromatography (FCC) was used to isolate monodisperse oligomeric species derived from oligo(CLs) (Table 2).

**Table 2:** Monodisperse oligomeric species isolated by flash column chromatography (FCC) and derived from ε-caprolactone (CL) using different types of aliphatic alcohols as initiators

No.	Sample	Initiator	Species	MW(theo) (g mol^−1^)	*M*_n_(NMR)	*T*_g_ a (°C)	*T*_m_ a (°C)
1	C_10_G-CL_1_	Geraniol	Monomer	268.39	257.22	−38	—
2	C_10_G-CL_2_	Geraniol	Dimer	382.53	397.36	−45	—
3	C_10_G-CL_3_	Geraniol	Trimer	496.67	570.86	−51	—
4	C_10_N-CL_1_	Nerol	Monomer	268.39	255.83	−39	—
5	C_10_N-CL_2_	Nerol	Dimer	382.53	390.52	−48	—
6	C_10_N-CL_3_	Nerol	Trimer	496.67	572.64	−50	—
7	C_10_C-CL_1_	β-Citronelol	Monomer	270.41	282.96	−38	—
8	C_10_C-CL_2_	β-Citronelol	Dimer	384.55	389.11	−45	—
9	C_10_C-CL_3_	β-Citronelol	Trimer	498.69	516.95	−50	—
10	C_15_F-CL_1_	Farnesol	Monomer	336.51	337.65	−27	—
11	C_15_F-CL_2_	Farnesol	Dimer	450.65	454.07	−36	—
12	C_15_F-CL_3_	Farnesol	Trimer	564.79	613.09	−38	—
13	C_15_1P-CL_1_	1-Pentadecanol	Monomer	342.56	347.11	—	43
14	C_15_1P-CL_2_	1-Pentadecanol	Dimer	456.70	474.95	—	34
15	C_15_1P-CL_3_	1-Pentadecanol	Trimer	570.84	583.38	—	36

a Obtained by differential scanning calorimetry (DSC), second scan.

In the case of the oligo(CL) functionalized with the olefinic end-group farnesyl (C_15_F-PCL; Table 1), a family of three different monodisperse oligomers was isolated after FCC: monomer (C_15_F-CL_1_), dimer (C_15_F-CL_2_), and trimer (C_15_F-CL_3_).

**Fig. 3:**
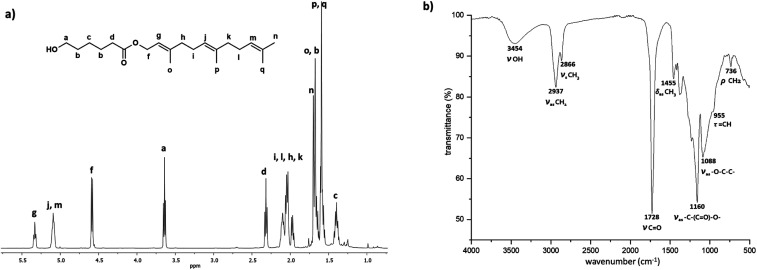
Spectra of a monomer C_15_F-CL_1_ (monodisperse oligomeric specie, Table 2) isolated by FCC from C_15_F-PCL (Table 1). (a) ^1^H NMR (500 MHz) in CDCl_3_, and (b) FTIR.

Different characterization techniques were used to illustrate the chemical nature of C_15_F-CL_1_. Fig. 3a shows the ^1^H NMR spectrum of C_15_F-CL_1_, which indicates characteristic peaks of both terminal groups, such as methylene adjacent to the hydroxyl group [*a*, –CH_2_–OH, *δ* 3.64] and methine of the vinyl groups in the farnesyl group [*g*, *j*, *m*, –(CH_3_)C=CH–, *δ* 5.33, 5.09].

The signals attributed to two methines showed an overlap of two triplets (*j* and *m*), where the relative ratio of *j* and *m* with respect to the third methine (*g*) is 2 : 1. A ratio of 2 : 2 was obtained for the two signals assigned to methylenes attached to the hydroxyl group (*a*) and ester terminal group [*f*, –CH_2_–O–(C=O)–, *δ* 4.58]. Thus, the integral values of both end-groups confirm the presence of C_15_F-CL_1_ as a monomer species.

Additionally, the ^1^H NMR spectrum of the monodisperse species C_15_F-CL_1_ (Fig. 3a) lacks the characteristic signal of the ester group [*e*, –CH_2_–O–(C=O)–, *δ* 4.05] in the repetitive unit of a typical oligomer such as C_15_F-PCL (Fig. 2). On the other hand, as shown in Fig. 3b, the Fourier-transform infrared (FTIR) spectrum of C_15_F-CL_1_ contained a band at 3454 cm^−1^, which was attributed to the hydroxyl group, as well as a band characteristic of ester carbonyl (C=O) at 1728 cm^−1^, another stretching vibration at 1160 cm^−1^ corresponding to the ester group [–(C=O)–O–], and a band at 955 cm^−1^ that was attributable to olefinic (C=CH) group vibration. The NMR and FTIR results suggest that C_15_F-CL_1_ is a monodisperse species. To confirm this, a mass spectrometry analysis was carried out.

**Fig. 4:**
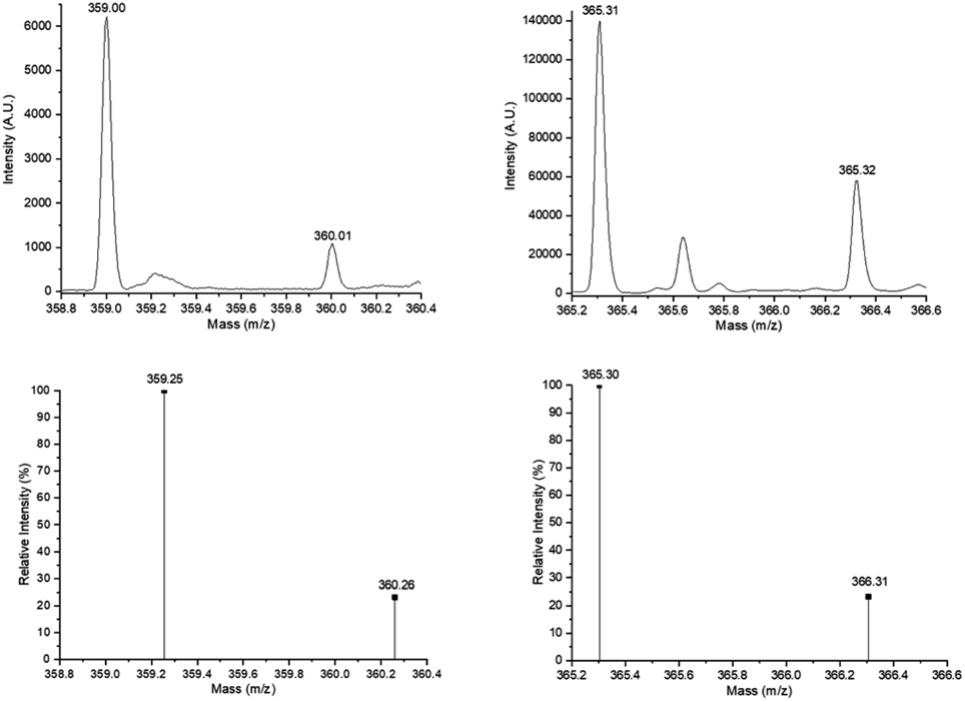
Experimental ESI/MS-QTOF spectrum in positive ionization mode of the monomer species [C_15_F-CL_1_Na^+^ (top, left) (Fig. 3a) and C_15_1P-CL_1_Na^+^ (top, right) (Fig. 7a)] and isotopic distribution calculated for DP = 1 [C_21_H_36_O_3_Na^+^ (C_15_F-CL_1_Na^+^, bottom, left) and C_21_H_42_O_3_Na^+^ (C_15_1P-CL_1_Na^+^, bottom, right)] in ref. 62 and 63http://www.chemcalc.org.^64^

Fig. 4 (top, left) shows the electrospray ionization quadrupole time-of-flight mass spectrometry (ESI/MS-QTOF) spectrum of the monodisperse monomer C_15_F-CL_1_ (Table 2) in positive mode after doping with sodium (Na^+^). The results confirmed the expected molecular weight. Compared with simulated spectrum, the difference was less than 0.30 g mol^−1^, indicating the presence of this monodisperse species.

**Fig. 5:**
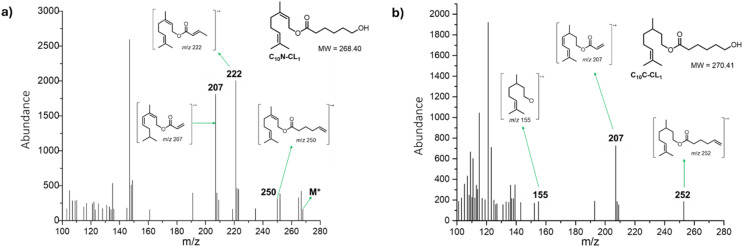
Electronic impact (EI) mass spectra (70 eV) of (a) C_10_N-CL_1_ and (b) C_10_C-CL_1_.

Other monodisperse monomers initiated by nerol (C_10_N-CL_1_) and citronellol (C_10_C-CL_1_) and isolated using FCC were analyzed by electron impact (EI) mass spectra (Fig. 5). In the mass spectrum in Fig. 5a, the molecular ion of C_10_N-CL_1_ at *m*/*z* 268 was observed, but there was a peak at *m*/*z* 250 corresponding to M-18 from loss of water, as well as a peak at *m*/*z* 222 resulting from allylic cleavage from the peak at *m*/*z* 250. The rationalized structure corresponding to the peak at *m*/*z* 207 is formed by double bond isomerization, resulting in increased conjugation. Similarly, the EI mass spectrum confirmed the presence of monodisperse species of C_10_C-CL_1_ (Fig. 5b). The peak at *m*/*z* 252 corresponds to the dehydration product, and the peak at *m*/*z* 207 is formed by dehydrogenation that increases the system conjugation and partial loss of the alkyl chain on the caprolactone side. Finally, the peak at *m*/*z* 155 corresponds to citronellol with one less proton.

**Fig. 6:**
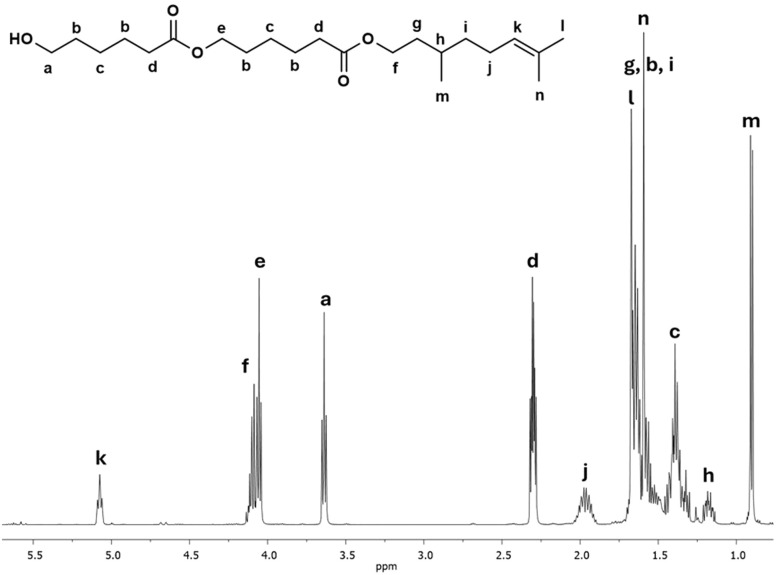
^1^H NMR (500 MHz) spectrum of a dimer derived from β-citronellol as initiator C_10_C-CL_2_ (monodisperse specie, Table 2) in CDCl_3_ isolated by FCC from C_10_C-PCL (Table 1).

Another interesting species in the sequences of the DP is a dimer derived from β-citronellol and 2-CL called C_10_C-CL_2_. Fig. 6 shows the ^1^H NMR spectrum of the C_10_C-CL_2_ isolated from the oligomer synthesized with β-citronellol (C_10_C-PCL).

The characteristic peaks show the presence of dimeric species, such as methylene adjacent to the hydroxyl group [*a*, –CH_2_–OH, *δ* 3.64], a signal for the vinyl group of the terpene [*k*, =CH–, *δ* 5.08], and a *d* signal composed of two triplets at the same chemical shift indicating two α-methyles into the dimer. Additionally, a new signal *e* is observed, which indicates the presence of a second monomeric unit of CL. This signal was is absent in the previous ^1^H NMR spectrum of the monodisperse monomer of C_15_F-CL_1_ (Fig. 3a). The methylene groups of the signals *a* and *d* [–CH_2_–(C=O)–O–, *δ* 4.10] adjacent to the ester group had a relative ratio of 2 : 4, which is evidence of a dimer species.

It is well known in organic and polymer chemistry that aliphatic and olefinic groups have different properties. Therefore, aliphatic and olefinic groups were compared as parts of terminal groups in a monodisperse species. The main idea was to contrast aliphatic and olefinic terminal groups with the same number of carbons (C_15_). Using a similar procedure to that described previously (Experimental section), a family of monodisperse species was derived from CL and functionalized with an aliphatic terminal group such as a pentadecyl (C_15_) group and isolated (C_15_-1P-CL_1_, C_15_-1P-CL_2_, and C_15_-1P-CL_3_, Table 2).

**Fig. 7:**
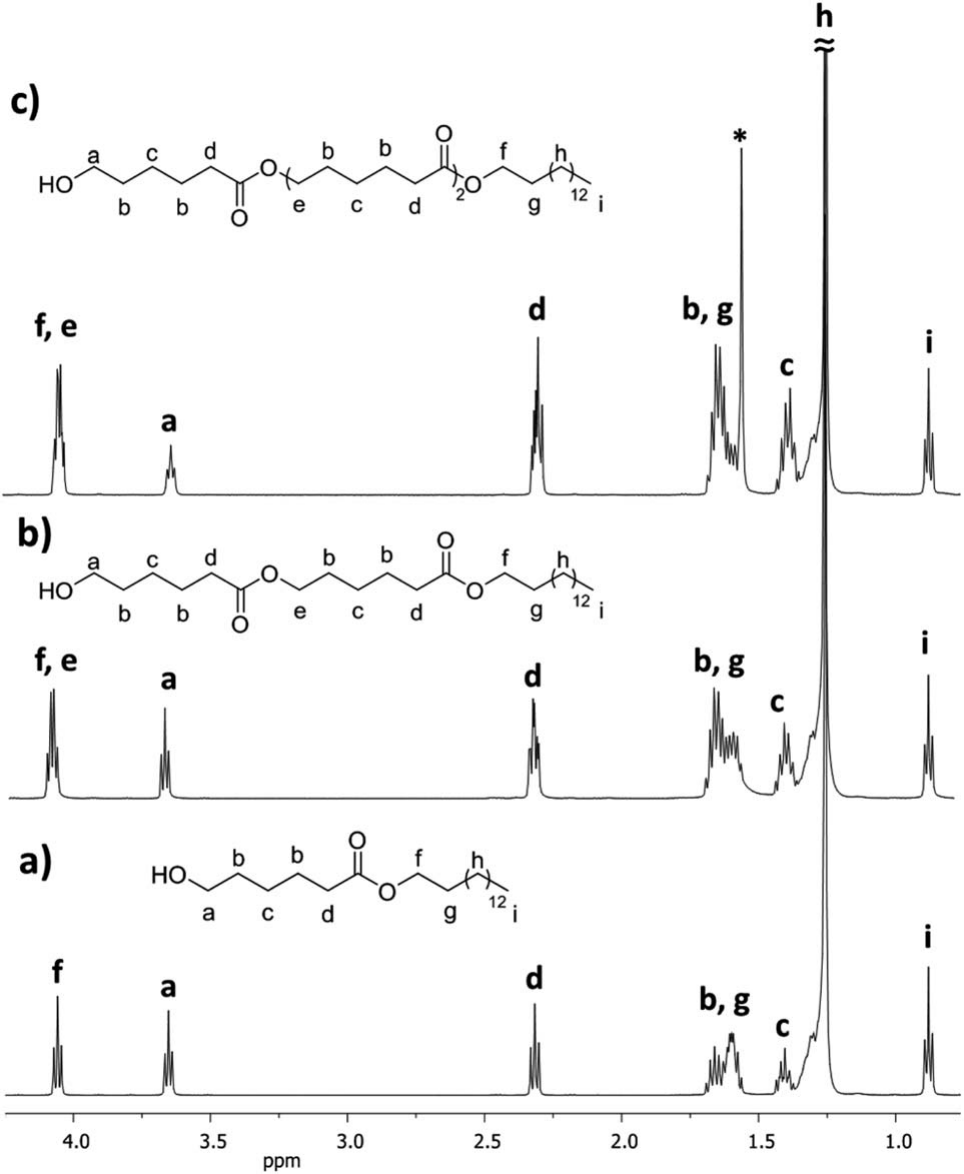
^1^H NMR (500 MHz) spectra in CDCl_3_ of the monodisperse oligomers derived from ε-caprolactone (CL) functionalized with 1-pentadecanol, isolated by flash column chromatography (FCC), (a) monomer C_15_1P-CL_1_, (b) dimer C_15_1P-CL_2_, and (c) trimer C_15_1P-CL_3_ (Table 2) (* signal of the water).

Fig. 7 shows ^1^H NMR spectra of the monomer (C_15_-1P-CL_1_), dimer (C_15_-1PCL_2_), and trimer (C_15_-1PCL_3_) functionalized with 1-pentadecyl. There were characteristic peaks of two terminal groups: the methyl group of the C_15_ moiety [*i*, CH_3_, *δ* 0.89] and the methylene adjacent to the hydroxyl group [*a*, CH_2_, *δ* 3.66].

The relative ratio of terminal groups *i* to *a* was 3 : 2. Additionally, the proportion of signals between *a* and methylene close to ester [*f* and *e*, CH_2_, *δ* 4.10] in the monomer (*a* to *f*; Fig. 7a), dimer (*a* to *f*,*e*; Fig. 7b) and trimer (*a* to *f*,*e*; Fig. 7c) was 2 : 2, 2 : 4, and 2 : 6, respectively, confirming the monodisperse oligomers. The ^13^C NMR spectrum also confirmed both terminal groups of C_15_1P-CL_1_, α-hydroxyl (*δ* 62.7 ppm, *a*) and ω-methyl (*δ* 22.6 ppm, *n*) (Fig. S12†). Fig. 4 (top, right) shows the ESI/MS-QTOF spectrum used to validate the chemical nature of C_15_-1P-CL_1_ by another technique. The theoretical and experimental signals correspond to the monomer functionalized with a pentadecyl terminal group doped with sodium (Na^+^). Three different monodisperse species (monomer, dimer, and trimer) were derived with an olefinic farnesyl end-group, isolated, and characterized. The monomer was previously illustrated in Fig. 3.

**Fig. 8:**
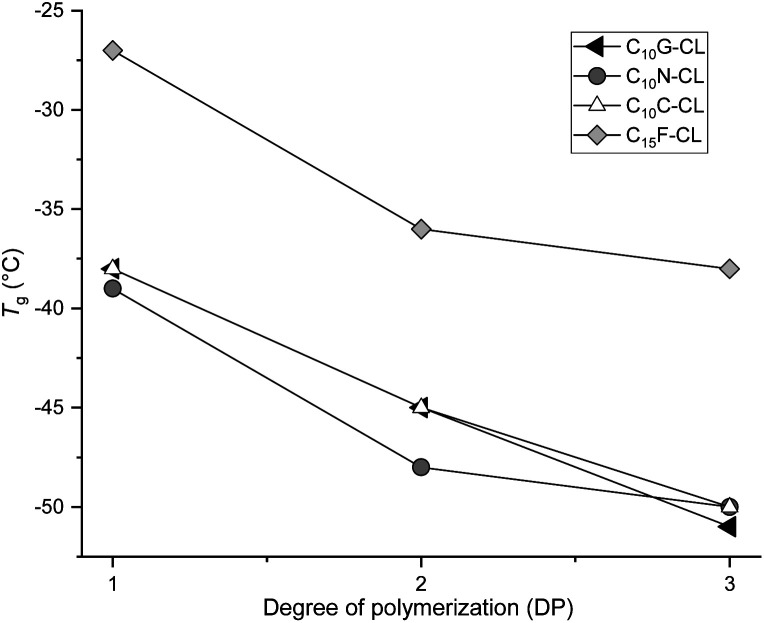
Plots of the family of monodisperse oligo(CL) with olefinic end-group. Effect of DP on the glass transition temperature (*T*_g_).

**Fig. 9:**
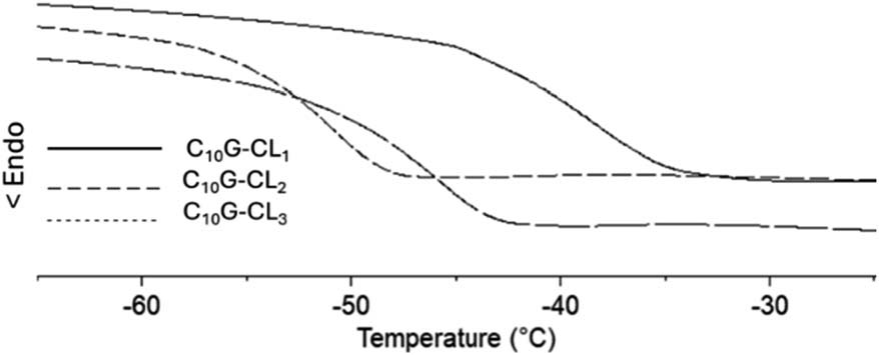
DSC thermograms [glass transition temperature (*T*_g_)] for species derived from monodisperse oligo(CL) from geraniol: monomer (C_15_G-CL_1_), dimer (C_15_G-CL_2_) and trimer (C_15_G-CL_3_).

Table 2 shows the thermal properties of the monodisperse species. No melting point (*T*_m_) was observed for all oligomeric monodisperse species derived from CL and olefinic terpenes (no. 1–12), indicating that the samples are amorphous with a liquid translucent appearance and viscosity. However, the glass transition temperature (*T*_g_) was observed in the entire family of oligoesters with the olefinic end-group (Fig. 8) with a characteristic pattern from monomer to trimer. In the case of oligomers with C_10_ (geraniol) terminal group (Fig. 9), *T*_g_ decreased from −38 (monomer) to −51 °C (trimer), indicating that the olefinic and methyl branch of the terminal group induced a disruption of order in the monodisperse oligo(CL) chain. This effect increased with the DP, with increases of DP producing an amorphous domain with an olefinic end-group. The same effect was observed for the rest of the family of monodisperse oligo(CLs) with an olefinic terminal group (Table 2, no. 1–12).

**Fig. 10:**
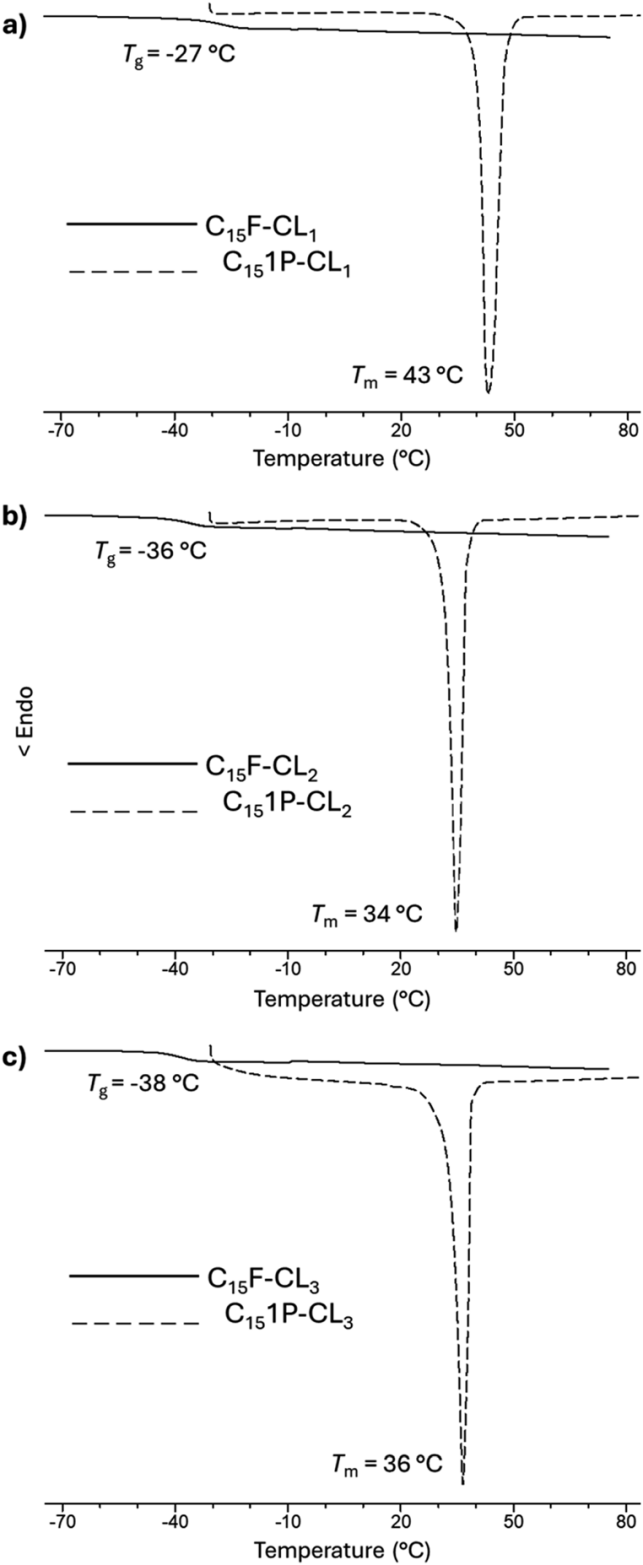
DSC thermograms for species derived from farnesol (C_15_F-CL_*x*_) and 1-pentadecanol (C_15_1P-CL_*x*_) (where *x* = 1, 2, 3): (a) monomer, (b) dimer and (c) trimer.

Monodisperse species with an aliphatic pentadecyl (C_15_) end-group formed a unique family that exhibited a melting temperature (*T*_m_). The monomer (C_15_-1P-CL_1_), dimer (C_15_-1P-CL_2_), and trimer (C_15_-1P-CL_3_) had *T*_m_ values of 34 to 43 °C. Olefinic and aliphatic terminal groups had an opposite effect on the PCL monodisperse oligomers. To illustrate both effects, Fig. 10 shows the DSC thermograms of two types of families with different end-groups (aliphatic C_15_-1P-CL and olefinic C_15_F-CL).

An amorphous domain was induced by the olefinic end-group farnesyl, and the semicrystalline domain was favored by the aliphatic terminal group pentadecyl. Both terminal groups induced monodisperse oligomers (monomer, dimer, and trimer) with the same physical properties as those of the alcohols or initiators [farnesol (C_15_H_26_O, liquid), and 1-pentadecanol (C_15_H_32_O, solid)].

**Fig. 11:**
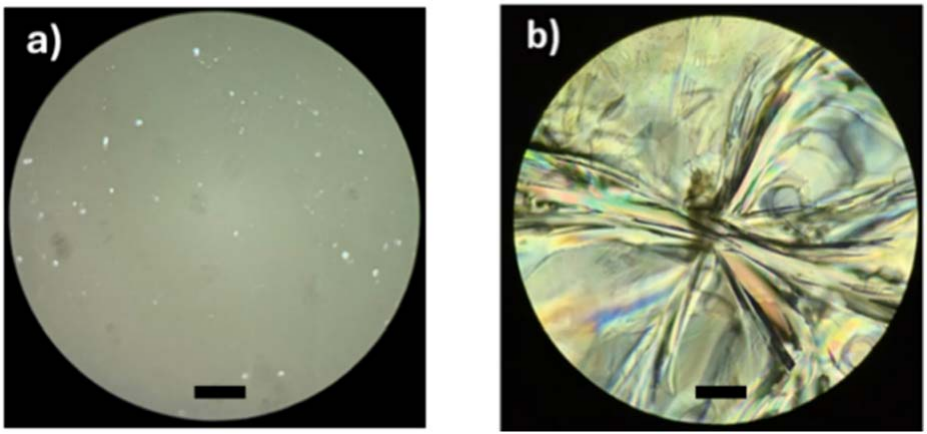
Polarized optical microscopy (POM, magnification: 40×) of (a) C_15_F-CL_1_ and (b) C_15_1P-CL_1_ (Table 2). Black bar = 50 μm.

These results suggest the importance of the alcohols (R-OH) as initiators and that the hybridization (sp^2^, olefin *vs.* sp^3^, aliphatic) and the substituents (methyl branch, olefin *vs.* linear, aliphatic) are the key to the physical properties of monodisperse monomer, dimer, and trimer species. To illustrate the physical properties of both monodisperse monomers with different terminal groups (C_15_F-CL_1_ and C_15_1P-CL_1_), Fig. 11 shows the POM micrography results obtained in different environments. C_15_F-CL_1_ appears as an amorphous liquid, but C_15_1P-CL_1_ exhibits a spherulite showing a semicrystalline domain.

**Fig. 12:**
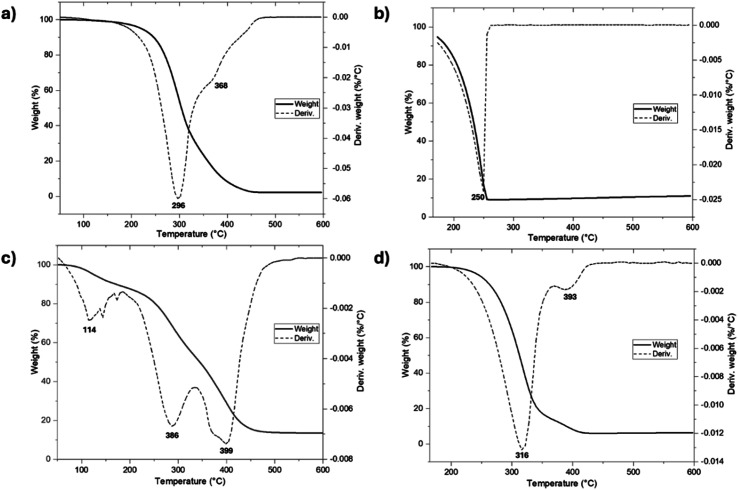
Thermal degradation (TGA) of (a) farnesol, (b) 1-pentadecanol, (c) C_15_F-CL_1_, and (d) C_15_1P-CL_1_.

To examine the thermal stability, the initiators were characterized by thermogravimetric analysis (TGA) (Fig. 12a and b). The thermograms show differences in the thermal stability between farnesol and 1-pentadecanol with a very different thermal decomposition temperatures (*T*_d_) of 296 and 250 °C, respectively. However, the monodisperse monomers functionalized with farnesyl (C_15_F-CL_1_) and pentadecyl (C_15_1P-CL_1_) end-groups (Fig. 12c and d) showed an increase in *T*_d_ relative to their initiators. Thus, the addition of a caprolactone unit provides an effect of thermal stability toward pyrolysis.

### 3.3. Biological properties of monodisperse oligo(CL)

**Fig. 13:**
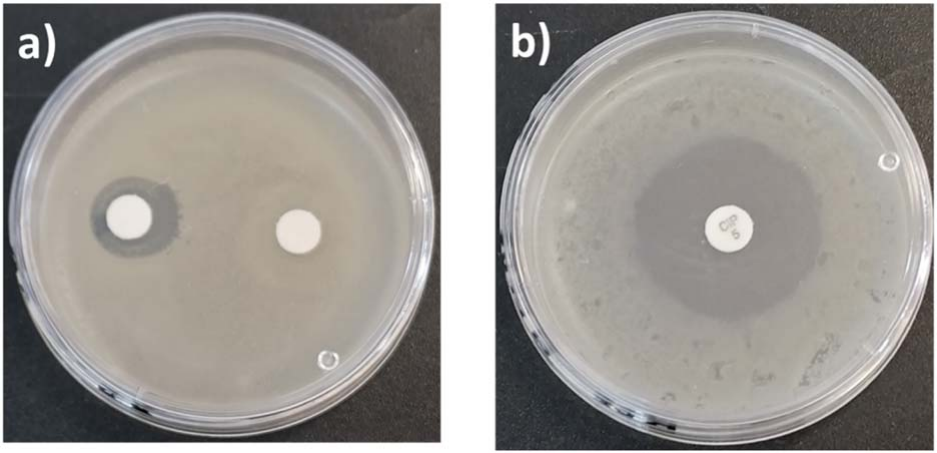
Antibiograms of *S. aureus* ATCC # 6538 in presence of (a) C_10_C-CL_1_ at 33 mg mL^−1^ (left), and 512 μg mL^−1^ (right). (b) Ciprofloxacin at 0.005 mg mL^−1^.

Terpenes such as β-citronellol, geraniol, nerol, and farnesol, have been studied due to their antibacterial activities.^34,39,64–70^ One of the goals in this work was to preserve the biological effects and properties of terpenes as part of terminal groups in monodisperse oligomeric species. First, a conventional antibiotic, ciprofloxacin, and a monodisperse oligomer, C_10_C-CL_1_, were compared using antibiograms (Fig. 13). There were dramatic differences in antibacterial activity and effective concentrations between ciprofloxacin and C_10_C-CL_1_. The C_10_C-CL_1_ inhibited the growth of *S. aureus* (Gram positive), but the inhibition decreased at low concentration.

The minimum inhibitory concentration (MIC) and minimum bactericidal concentration (MBC) were studied using the NCCLS method^71^ and two types of bacteria: *S. aureus* (Gram positive) and *P. aeruginosa* (Gram negative) (Table 3). All alcohols previously used as initiators in the polymerizations (β-citronellol, nerol, geraniol, farnesol, and 1-pentadecanol) showed antibacterial activity, as reported previously.^34,39,64–70^

**Table 3:** MIC and MBC values in μg mL^−1^ obtained after 18 h of contact for initiators and monodisperse oligomeric species, according with the CLSI M07 method

		*S. aureus* ATCC #6538		*P. aeruginosa* ATCC # 13388	
		MIC (95%)	MBC	MIC (95%)	MBC
Reference	Ciprofloxacin	0.125 ± 0.01	0.25 ± 0.01	≤0.06 ± 0.01	0.06 ± 0.01
Series 1	Farnesol	32 ± 0.01	64 ± 0.01	64 ± 0.01	128 ± 0.01
	C_15_F-CL_1_	64 ± 0.01	128 ± 0.02	64 ± 0.05	128 ± 0.05
	C_15_F-CL_2_	64 ± 0.02	128 ± 0.02	64 ± 0.01	128 ± 0.01
	C_15_F-CL_3_	64 ± 0.05	128 ± 0.05	64 ± 0.06	128 ± 0.06
Series 2	Nerol	64 ± 0.02	128 ± 0.02	32 ± 0.01	64 ± 0.01
	C_10_N-CL_1_	32 ± 0.01	64 ± 0.01	64 ± 0.05	128 ± 0.05
	C_10_N-CL_2_	64 ± 0.05	128 ± 0.05	64 ± 0.05	128 ± 0.05
	C_10_N-CL_3_	64 ± 0.05	≥256 ± 0.06	64 ± 0.05	128 ± 0.05
Series 3	β-Citronellol	32 ± 0.02	64 ± 0.01	64 ± 0.04	128 ± 0.05
	C_10_C-CL_1_	64 ± 0.05	256 ± 0.05	32 ± 0.01	64 ± 0.01
	C_10_C-CL_2_	32 ± 0.02	64 ± 0.01	32 ± 0.02	64 ± 0.01
	C_10_C-CL_3_	32 ± 0.01	64 ± 0.01	64 ± 0.05	128 ± 0.05
Series 4	Geraniol	64 ± 0.06	256 ± 0.05	32 ± 0.04	64 ± 0.01
	C_10_G-CL_1_	64 ± 0.04	≥256 ± 0.05	64 ± 0.02	128 ± 0.01
	C_10_G-CL_2_	64 ± 0.02	≥256 ± 0.05	64 ± 0.05	128 ± 0.01
	C_10_G-CL_3_	32 ± 0.01	≥256 ± 0.05	64 ± 0.06	128 ± 0.01
Series 5	1-Pentadecanol	64 ± 0.04	≥256 ± 0.05	32 ± 0.01	64 ± 0.04
	C_15_1P-CL_1_	64 ± 0.04	≥256 ± 0.06	16 ± 0.01	32 ± 0.01
	C_15_1P-CL_2_	64 ± 0.02	≥256 ± 0.05	32 ± 0.04	64 ± 0.05
	C_15_1P-CL_3_	64 ± 0.04	≥256 ± 0.05	32 ± 0.02	64 ± 0.05

In the case of *S. aureus*, an MIC of 32 μg mL^−1^ was obtained for β-citronellol and farnesol. The same result was found for geraniol, nerol, and 1-pentadecanol with *P. aeruginosa*.

The antimicrobial activity of terpenes against Gram-positive bacteria is influenced by their lipophilicity and hydrophobicity, as well as the presence of hydroxyl groups.^70,72^ The bactericidal behavior of oligomers can be regulated through the incorporation of terpene alcohols, which modulate the membrane stability of *S. aureus* or potentiate their bacterial loss integrity.^73^ According to Lopez-Romero *et al.*,^74^ the bactericidal mechanisms of essential oils such as β-citronellol include surface-charge alteration and K^+^ leakage, which improve the disruption of *S. aureus* membranes.

**Fig. 14:**
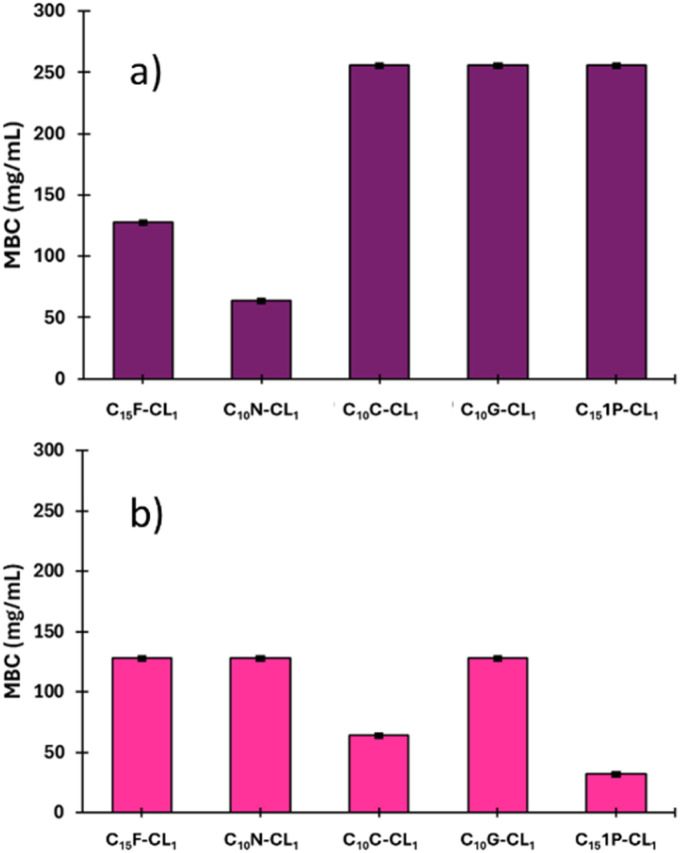
Effect of monodisperse oligomeric (monomer, CL_1_) species on the minimum bactericidal concentration (MBC) of two types of bacteria: (a) *S. aureus* (Gram positive) and (b) *P. aeruginosa* (Gram negative).

**Fig. 15:**
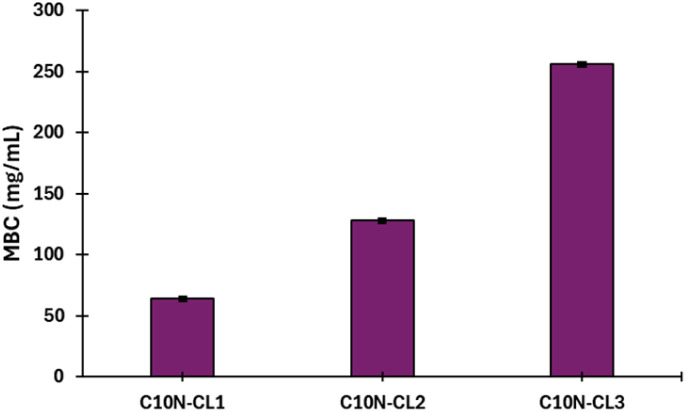
Effect of the monodisperse oligomeric species with nerol as terminal group on the minimum bactericidal concentration (MBC) of *S. aureus* (Gram positive). Monomer [C_10_N-CL_1_, nerol wt% = 57], dimer [C_10_N-CL_2_, nerol wt% = 40], and trimer [C_10_N-CL_3_, nerol wt% = 31].

As shown in Table 3, the monodisperse oligomers functionalized with terpenes had antibacterial activity and preserved properties similar to those of their precursors. For instance, the length of the oligomers (C_10_ or C_15_) and the unsaturation degree are independent of the bactericidal response, which is mainly attributed to each terpene as terminal group. However, higher bactericidal response is observed in specific monomers, including C_10_N-CL_1_, and C_10_C-CL_2–3_ against *S. aureus*, and C_10_1P-CL_1_ against *P. aeruginosa* (Fig. 14). This behavior indicates that the size of monodisperse oligomers, for example, mainly monomers plays a crucial role in the antibacterial properties associated with the availability of the terminal chain of terpenes, which can perform the ionic imbalance through the cell membrane and interfere with glycan synthesis, resulting in cell death.^75,76^ For example, for series 1, the results with *S. aureus* showed that farnesol had half the values of MIC (32 μg mL^−1^) and MBC (64 μg mL^−1^) with respect to the monomer (C_15_F-CL_1_), dimer (C_15_F-CL_2_), and trimer (C_15_F-CL_3_). This indicated a decreased activity of oligomers, which is probably due to the repetitive unit of CL. However, in the case of *P. aeruginosa*, farnesol and the oligomers exhibited an MIC and MBC of 64 μg mL^−1^ and 128 μg mL^−1^, respectively. C_15_F-CL_1_, C_15_F-CL_2_, and C_15_F-CL_3_ exhibited the same antibacterial activity (MIC and MBC) for both *S. aureus* and *P. aeruginosa*. On the other hand, comparing two types of geometric isomers as terminal groups in terms of MBC, such as, derivatives of nerol [*Z* isomer or *cis*, (C_10_N-CL_*x*_)] *vs.* geraniol [*E* isomer or *trans*, (C_10_G-CL_*x*_)] (Table 3), the effect on the *P. aeruginosa* was negligible. However, with the bacterium *S. aureus* the case was dramatically different, where C_10_N-CL_*x*_ monodisperse species showed a pattern on the antibacterial activity, where monomer > dimer > trimer (Fig. 15). In contrast, C_10_G-CL_*x*_ species had non-significant effect. So, this result suggests that *Z* isomer (or *cis*) end group in the C_10_N-CL_*x*_ can induce a significant disruption, probably, in the cell membrane of *S. aureus*, and this effect is directly proportional to the weight percent (wt%) of the nerol as a terminal group, from trimer (31%) to monomer (57%) (Fig. 15). In this sense, the exploration of organic molecules with *Z* isomers (or *cis* isomers as end-groups) as a significant factor against *S. aureus* will be worked in a future contribution.

Although the monodisperse species (Table 3) demonstrated antibacterial activity, there was a significant gap from that of the conventional antibiotic ciprofloxacin, which has 512 times higher activity in terms of MIC for *S. aureus*. This is attributed to the direct inhibition of the DNA-gyrase and prevention of bacterial DNA replication. It is important to note that 1-pentadecanol oligomers demonstrate slight bactericidal behavior against *S. aureus*. However, the MIC value of C_15_1P-CL_1_ against *P. aeruginosa* is 16 μg mL^−1^ with the pentadecyl end-group. The key point was the difference with *P. aeruginosa*, for which MIC and MBC were low compared to *S. aureus*. These results suggest that an aliphatic terminal group tends to act more as an antibiotic on the Gram-negative bacterium such as *P. aeruginosa* compared to the Gram-positive bacterium *S. aureus*. Thus, the bacterial membrane composition (particularly peptidoglycan) plays a key role in the oligomer interaction.

It is well known that the molecules as terpenes and aromatic compound has been explored in terms of their antifungal^77,78^ and antibacterial^79^ activity, for example, nerol,^77^ citral,^78^*trans*-anethole^79^ and estragole.^79^ In these cases, the inherent hydrophobicity to the terpenes play a significant role, where the affinity and accumulation of those in the cell membrane produce a loss of membrane integrity;^79^ this phenome was detected with an increase in the extracellular conductivity and extracellular pH,^77,78^ which indicates rapid leakage of ions; complementary, using scanning electron microscopy (SEM) was observed severe effects on the cell wall and cytoplasmic membrane.^79^ In the case of monodisperse oligomers (Table 3), probably, the mechanism of antibacterial activity involves a significant membrane disruption against bacteria, however, more studies will be explored in our laboratory to validate the damage.

## 4. Conclusions

In this work, a series of oligomers derived from PCL were synthesized by ring-opening polymerization (ROP) of ε-caprolactone (CL) using terpenes as initiators called oligo(CLs). The oligo(CLs) had specific terminal groups derived from terpenes. Using a flash column chromatography (FCC) a family of fifteen monodisperse species such as monomer, dimer, and trimer were isolated. The thermal properties of monodisperse species derived from olefinic terpenes included a unique glass transition temperature (*T*_g_), which decreased from the monomer to the trimer and increased the amorphous domain. However, monodisperse oligomers derived from aliphatic pentadecyl exhibited a semicrystalline domain with characteristic melting temperature (*T*_m_).

There were remarkable differences in physical properties between monodisperse oligomeric species with farnesyl and pentadecyl end-groups, which both have the same number of carbons (C_15_) but different functionality. Farnesyl produced liquid oligomers, and pentadecyl produced semicrystalline powders. This is the first report of using a family of terpenes to functionalize monodisperse oligomeric species. Due to the antibacterial properties of terpenes and their use as initiators, the monodisperse species showed antibacterial activity, especially for Gram-positive *S. aureus*. These monodisperse species could lead to new antibiotic compounds with potential applications. Studies on the mechanisms of action are currently underway in our laboratory.

## Data availability

The data supporting this article have been included as part of ESI.†

## Author contributions

María Guadalupe Ortiz-Aldaco: investigation, validation, formal analysis, writing – original draft. Miriam Estévez: funding acquisition. Beatriz Liliana España-Sánchez: biological properties, supervision. José Bonilla-Cruz: thermal properties. Eloy Rodríguez-deLeón: mass spectrometry. José E. Báez: conceptualization, supervision, formal analysis, writing – original draft, writing – review, funding acquisition.

## Conflicts of interest

There are no conflicts to declare.

## Supplementary Material

RA-015-D4RA08104H-s001
