# Supplementary material for: Monodisperse oligo(ε-caprolactones) with terpenes and alkyl end-groups: synthesis, isolation, characterization, and antibacterial activity
Source: RSC Adv. 2025 Jan 2;15(1):276–88. doi: 10.1039/d4ra08104h (PMC11694720; doi:10.1039/d4ra08104h)
Supplement: RA-015-D4RA08104H-s001 [file RA-015-D4RA08104H-s001.pdf]

Supplementary Information (SI) for RSC Advances.
This journal is © The Royal Society of Chemistry 2024

Supplementary Information

Monodisperse oligo(ε-caprolactones) with terpenes and alkyl end-groups: synthesis, isolation, characterization, and antibacterial activity

María Guadalupe Ortiz-Aldaco,[a] Miriam Estévez,[b] Beatriz Liliana España-Sánchez,[c] José Bonilla-Cruz,[d] Eloy Rodríguez-deLeón,[e] and José E. Báez[a]*

[a] University of Guanajuato (UG), Department of Chemistry, Noria Alta S/N, Col. Noria Alta, Guanajuato, Gto. México, 36050.

[b] Universidad Nacional Autonoma de Mexico (UNAM), Centro de Física Aplicada y Tecnología Avanzada (CFATA), Boulevard Juriquilla 3001, Querétaro, Qro. México, 76230.

[c] Centro de Investigación y Desarrollo Tecnológico en Electroquímica, S.C. (CIDETEQ), Santiago de Queretaro, Qro. México, 76703.

[d] Centro de Investigación en Materiales Avanzados (CIMAV), Unidad Monterrey, Polymer Science, Alianza Norte 202, Nueva Autopista Monterrey-Aeropuerto Km 10, Apodaca, N.L. México, 66628.

[e] Autonomous University of Querétaro (UAQ), Posgrado en Ciencias Químico Biológicas, Faculty of Chemistry, Queretaro, Qro. México, 76010.

To whom correspondence should be addressed: José E. Báez, e-mail: jebaez@ugto.mx

Table of Contents

		Page
**Tables**		
	**Table S1** Hydrolytic degradation of initiators (farnesol and 1-pentadecanol), monodisperse monomers (C_{15}F-CL_1 and C_{15}1P-CL_1), and monodisperse dimers (C_{15}F-CL_2 and C_{15}1P-CL_2).	4
**Figures**		
	**Fig. S1** ^{1}H NMR (500 MHz) spectrum of geraniol (C_{10}) in $CDCl_3$ at 40 °C.	5
	**Fig. S2** ^{1}H NMR (500 MHz) spectrum of nerol (C_{10}) in $CDCl_3$ at 40 °C.	6
	**Fig. S3** ^{1}H NMR (500 MHz) spectrum of *β*-citronellol (C_{10}) in $CDCl_3$ at 40 °C.	7
	**Fig. S4** ^{1}H NMR (500 MHz) spectrum of farnesol (C_{15}) in $CDCl_3$ at 40 °C.	8
	**Fig. S5** ^{1}H NMR (500 MHz) spectrum of 1-pentadecanol (C_{15}) in $CDCl_3$ at 40 °C.	9
	**Fig. S6** ^{1}H NMR (500 MHz) spectrum of oligo(CL) synthetized using geraniol as initiator (C_{10}G-PCL, Table 1) in $CDCl_3$ at 40 °C.	10
	**Fig. S7** ^{1}H NMR (500 MHz) spectrum of oligo(CL) synthetized using *β*-citronellol as initiator (C_{10}C-PCL, Table 1) in $CDCl_3$ at 40 °C.	11
	**Fig. S8** ^{1}H NMR (500 MHz) spectrum of oligo(CL) synthetized using 1-pentadecanol as initiator (C_{15}1P-PCL, Table 1) in $CDCl_3$ at 40 °C.	12
	**Fig. S9** ^{1}H NMR (500 MHz) spectrum of a monomer derived from *β*-citronellol as initiator C_{10}C-CL_1 (monodisperse specie, Table 2) in $CDCl_3$ isolated by FCC from C_{10}C-PCL (Table 1).	13
	**Fig. S10** ^{1}H NMR (500 MHz) spectrum of a trimer derived from *β*-citronellol as initiator C_{10}C-CL_3 (monodisperse specie, Table 2) in $CDCl_3$ isolated by FCC from C_{10}C-PCL (Table 1).	14
	**Fig. S11** ^{1}H NMR (500 MHz) spectrum of a dimer derived from farnesol as initiator C_{15}F-CL_2 (monodisperse specie, Table 2) in $CDCl_3$ isolated by FCC from C_{15}F-PCL (Table 1).	15
	**Fig. S12** ^{13}C NMR (500 MHz) spectrum of a monomer derived from 1-pentadecanol as initiator C_{15}1P-CL_1 (monodisperse specie, Table 2) in $CDCl_3$ isolated by FCC from C_{15}F-PCL (Table 1).	16
	**Fig. S13** FT-IR spectrum and assignment of bands from monodisperse monomer C_{10}G-CL_1.	17
	**Fig. S14** FT-IR spectrum and assignment of bands from monodisperse dimer C_{10}G-CL_2.	18
	**Fig. S15** FT-IR spectrum and assignment of bands from	19

**Figures** (continuted)	monodisperse trimer $C_{10}G-CL_3$.	**20**
	**Fig. S16** FT-IR spectrum and assignment of bands from monodisperse monomer $C_{10}N-CL_1$.	**21**
	**Fig. S17** FT-IR spectrum and assignment of bands from monodisperse dimer $C_{10}N-CL_2$.	**22**
	**Fig. S18** FT-IR spectrum and assignment of bands from monodisperse trimer $C_{10}N-CL_3$.	**23**
	**Fig. S19** FT-IR spectrum and assignment of bands from monodisperse monomer $C_{10}C-CL_1$.	**24**
	**Fig. S20** FT-IR spectrum and assignment of bands from monodisperse dimer $C_{10}C-CL_2$.	**25**
	**Fig. S21** FT-IR spectrum and assignment of bands from monodisperse trimer $C_{10}C-CL_3$.	**26**
	**Fig. S22** FT-IR spectrum and assignment of bands from monodisperse dimer $C_{15}F-CL_2$.	**27**
	**Fig. S23** FT-IR spectrum and assignment of bands from monodisperse trimer $C_{15}F-CL_3$.	**28**
	**Fig. S24** FT-IR spectrum and assignment of bands from monodisperse monomer $C_{15}1P-CL_1$.	**29**
	**Fig. S25** FT-IR spectrum and assignment of bands from monodisperse dimer $C_{15}1P-CL_2$.	**30**
	**Fig. S26** FT-IR spectrum and assignment of bands from monodisperse trimer $C_{15}1P-CL_3$.	**31**
	**Fig. S27** Thermal degradation (TGA) of a) $C_{15}F-CL_2$ and b) $C_{15}1P-CL_2$.	

Table S1 Hydrolytic degradation of initiators (farnesol and 1-pentadecanol), monodisperse monomers ($C_{15}F-CL_1$ and $C_{15}1P-CL_1$), and monodisperse dimers ($C_{15}F-CL_2$ and $C_{15}1P-CL_2$).

Sample	T_{d1} (°C)	Weight loss (%)	T_{d2} (°C)	Weight loss (%)	T_{d3} (°C)	Weight loss (%)
Farnesol	296	42	368	83	—	—
1-pentadecanol	250	83	—	—	—	—
$C_{15}F-CL_1$	114	3	386	31	399	70
$C_{15}1P-CL_1$	316	54	393	91	—	—
$C_{15}F-CL_2$	132	2	298	23	410	67
$C_{15}1P-CL_2$	369	47	410	80	—	—

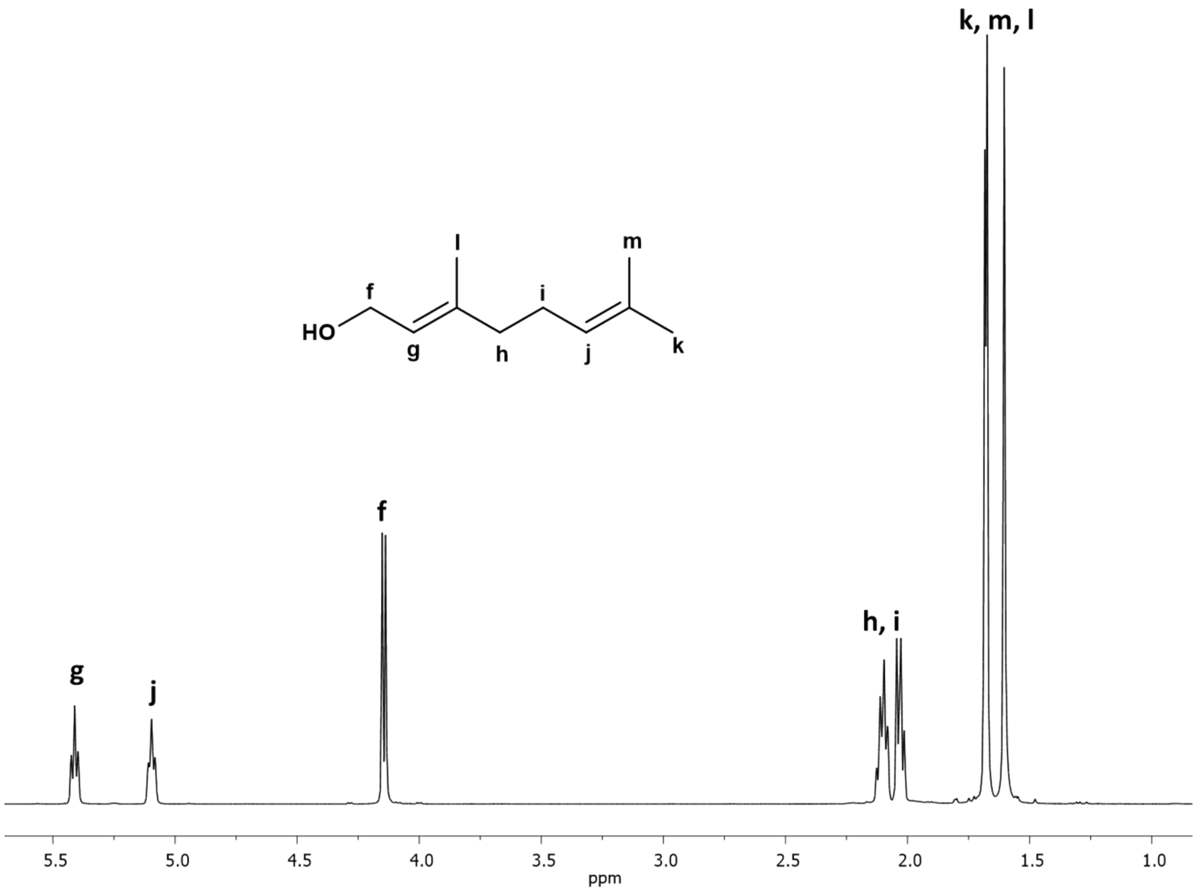

Fig. S1 ^{1}H NMR (500 MHz) spectrum of geraniol (C_{10}) in $CDCl_3$ at 40 °C.

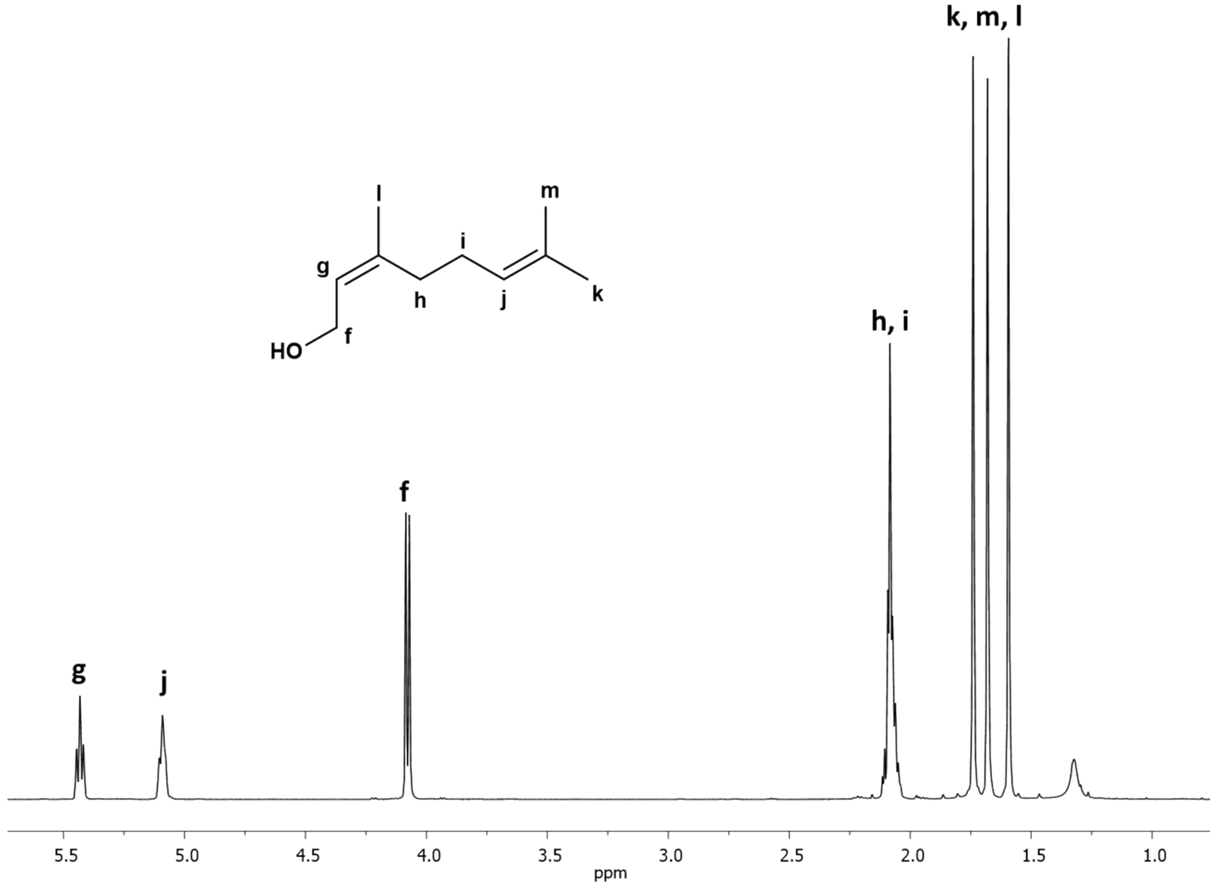

Fig. S2 ^{1}H NMR (500 MHz) spectrum of nerol (C_{10}) in $CDCl_3$ at 40 °C.

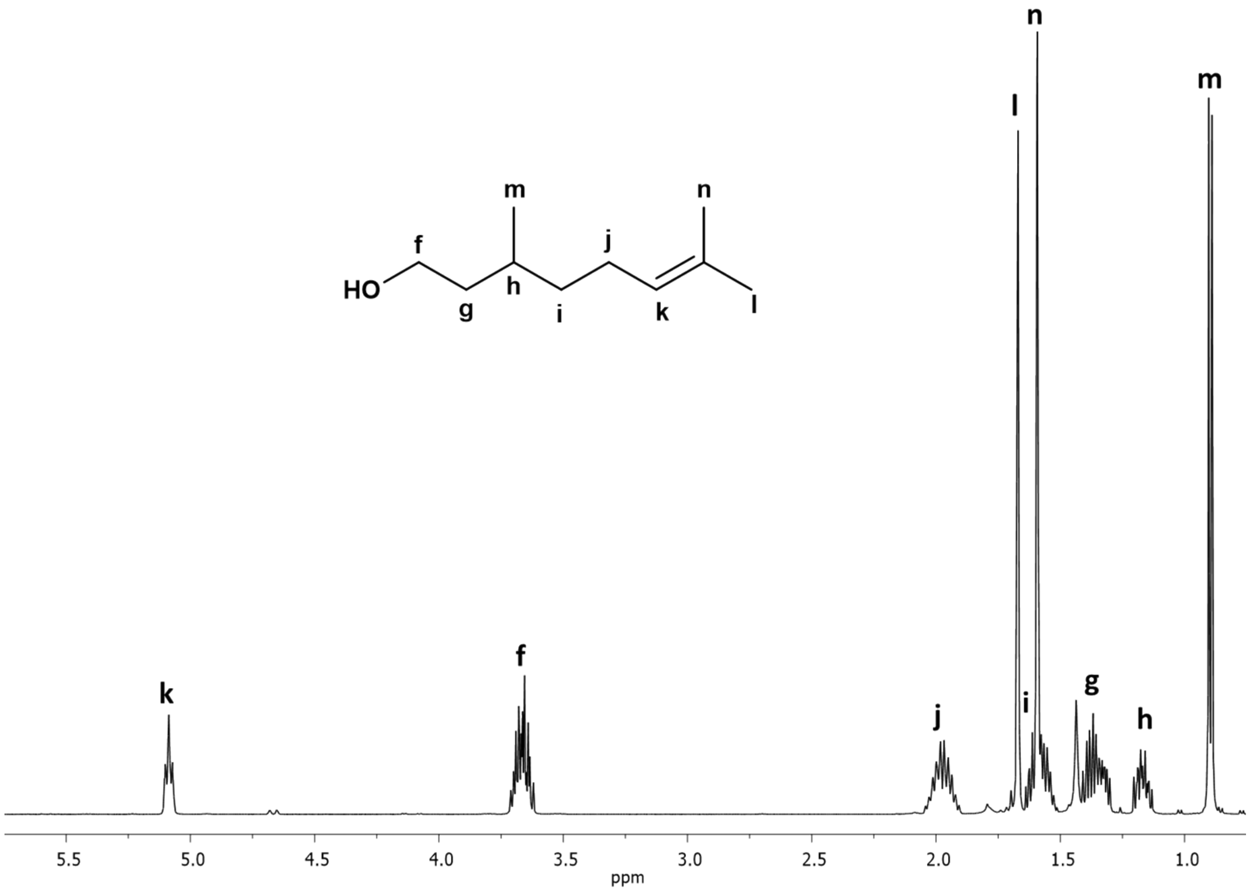

Fig. S3 ^{1}H NMR (500 MHz) spectrum of *β*-citronellol (C_{10}) in $CDCl_3$ at 40 °C.

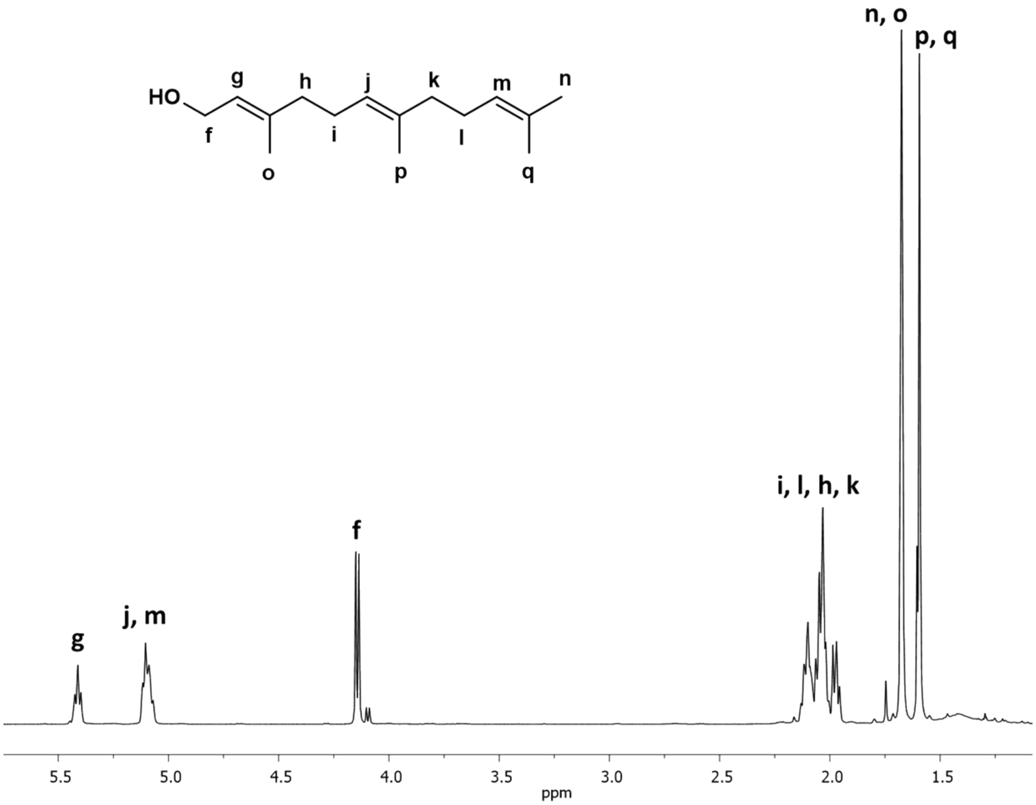

Fig. S4 ^{1}H NMR (500 MHz) spectrum of farnesol (C_{15}) in $CDCl_3$ at 40 °C.

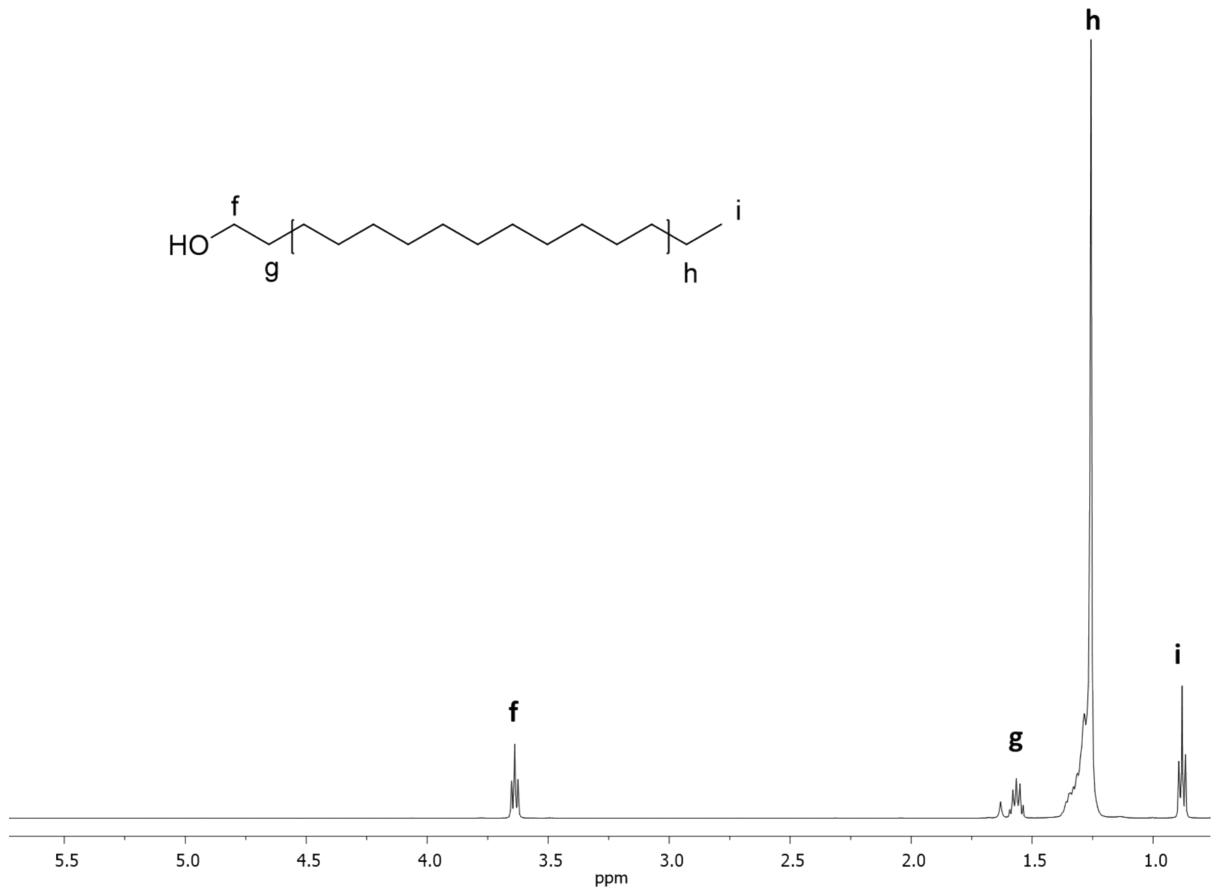

Fig. S5 ^{1}H NMR (500 MHz) spectrum of 1-pentadecanol (C_{15}) in $CDCl_3$ at 40 °C.

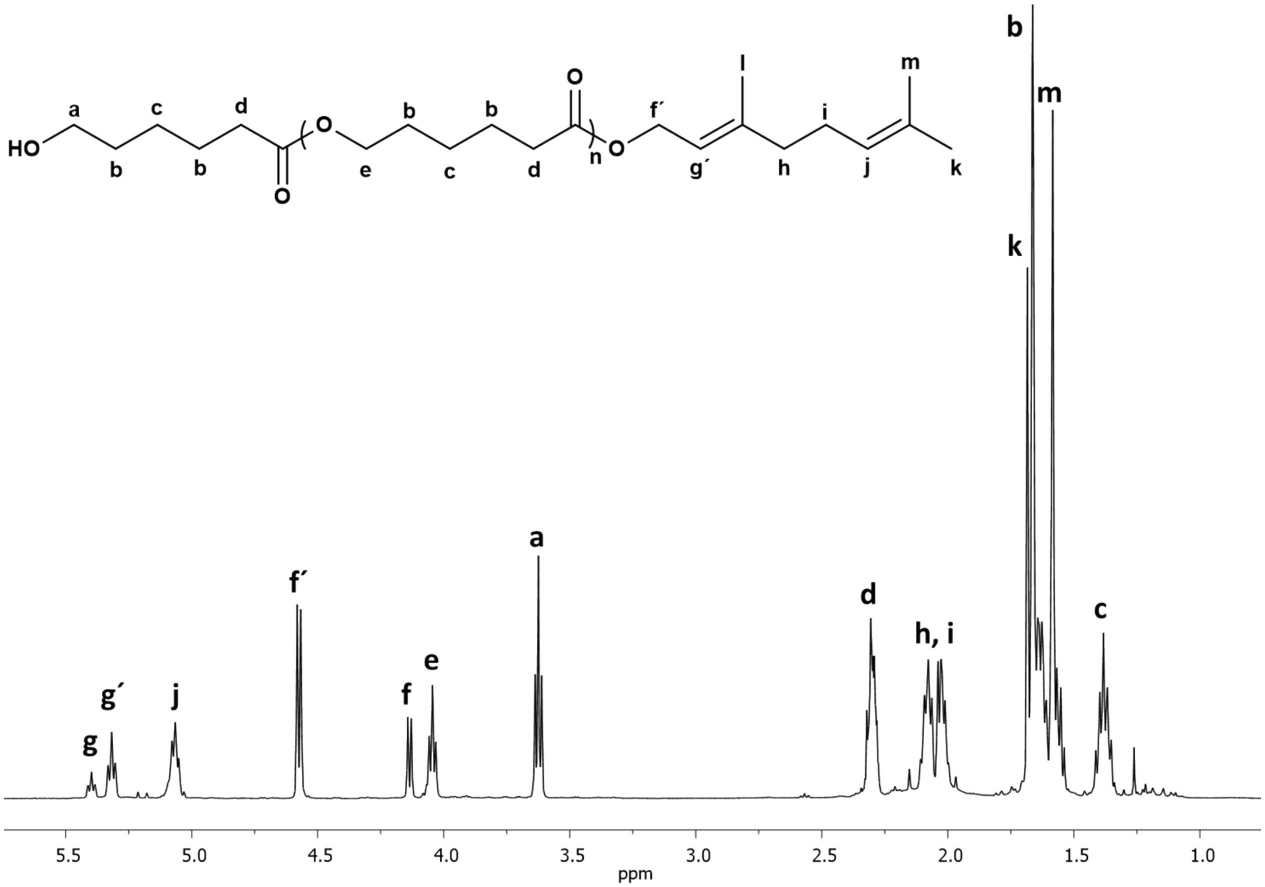

Fig. S6 ^{1}H NMR (500 MHz) spectrum of oligo(CL) synthetized using geraniol as initiator (C_{10}G-PCL, Table 1) in $CDCl_3$ at 40 °C.

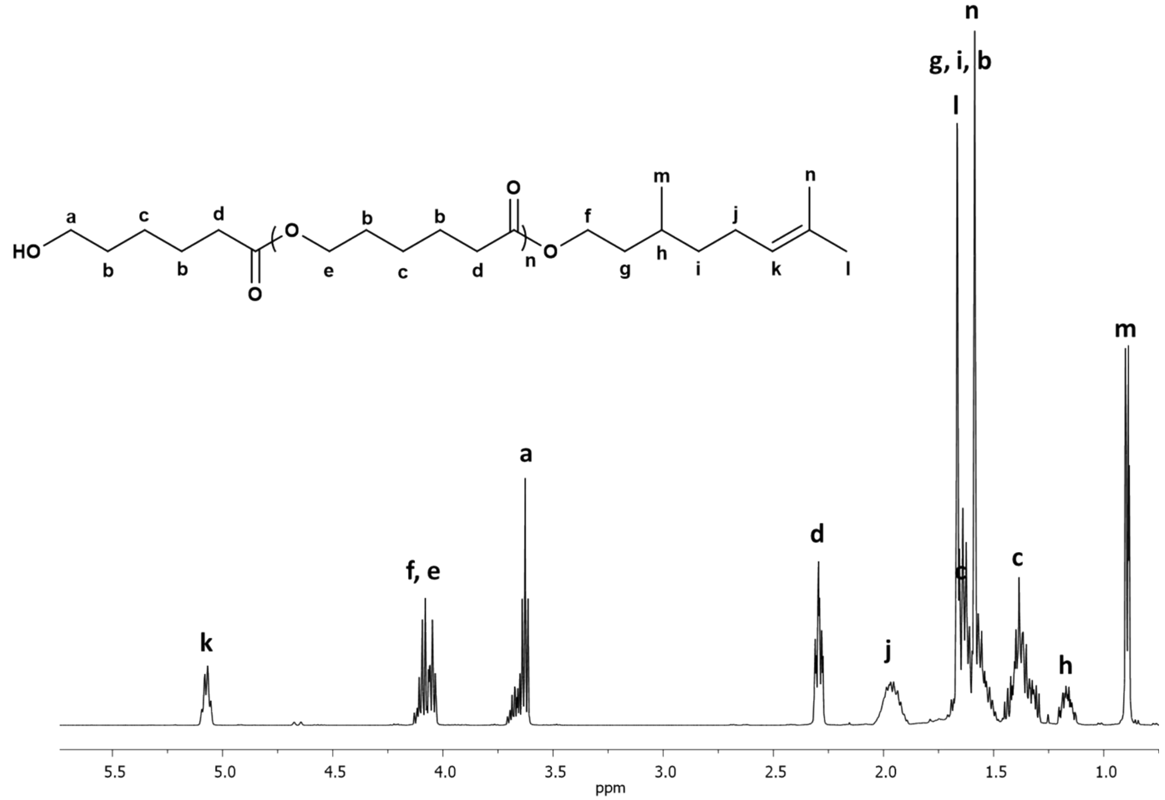

Fig. S7 ^{1}H NMR (500 MHz) spectrum of oligo(CL) synthetized using *β*-citronellol as initiator (C_{10}C-PCL, Table 1) in $CDCl_3$ at 40 °C.

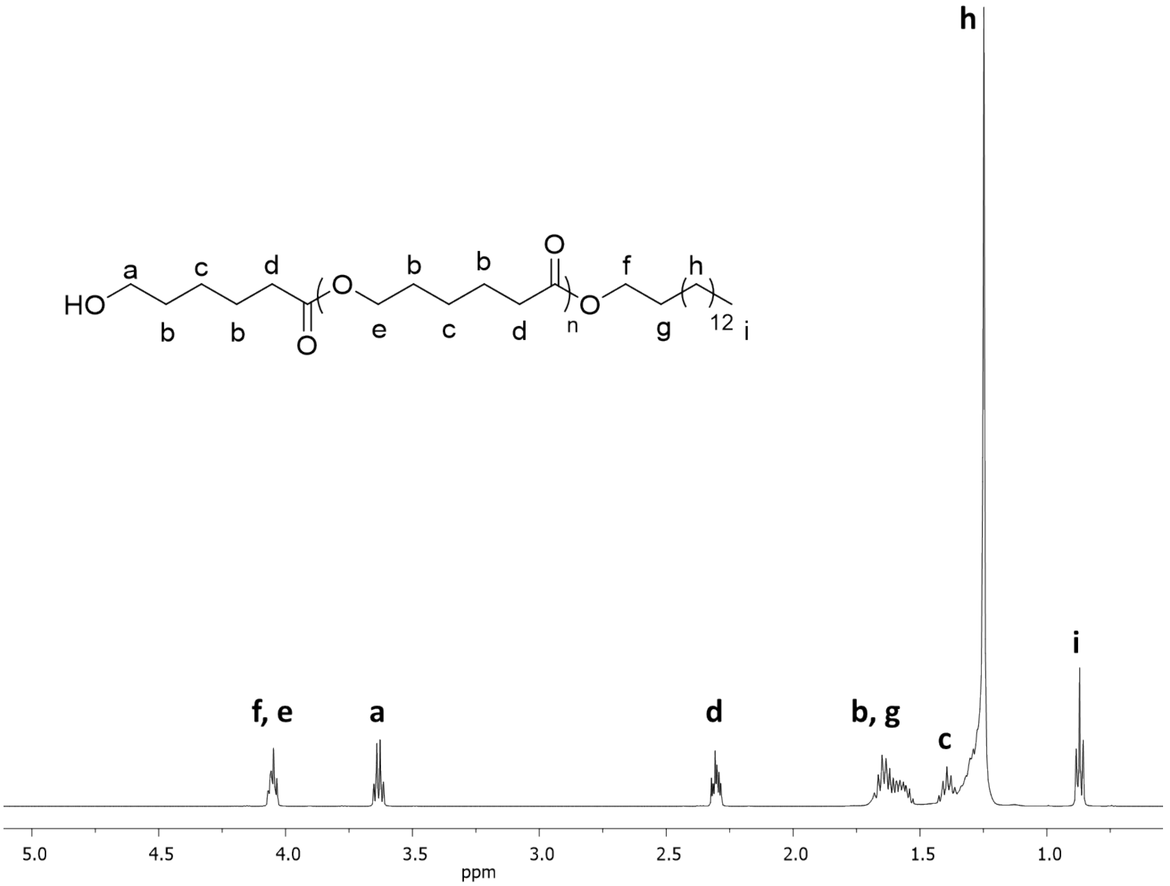

Fig. S8 ^{1}H NMR (500 MHz) spectrum of oligo(CL) synthetized using 1-pentadecanol as initiator (C_{15}1P-PCL, Table 1) in $CDCl_3$ at 40 °C.

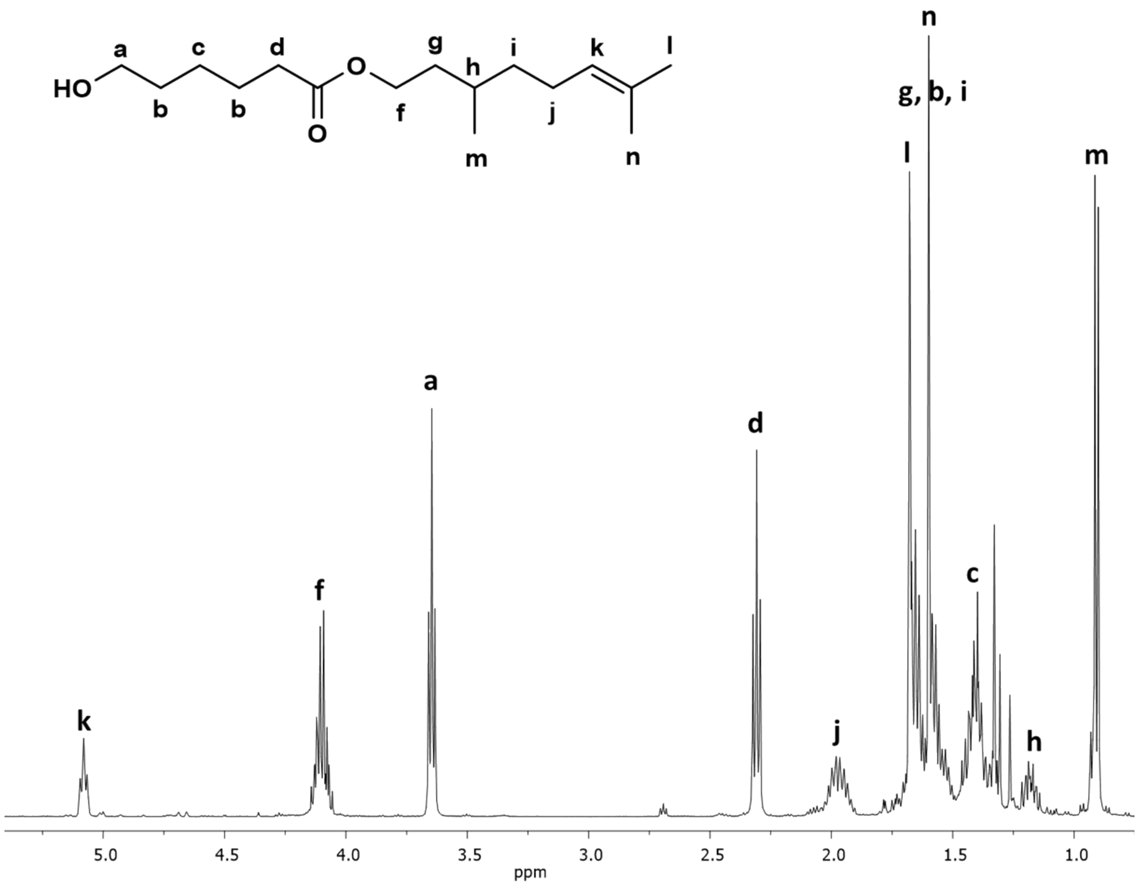

Fig. S9 ^{1}H NMR (500 MHz) spectrum of a monomer derived from *β*-citronellol as initiator C_{10}C-CL_1 (monodisperse specie, Table 2) in $CDCl_3$ isolated by FCC from C_{10}C-PCL (Table 1).

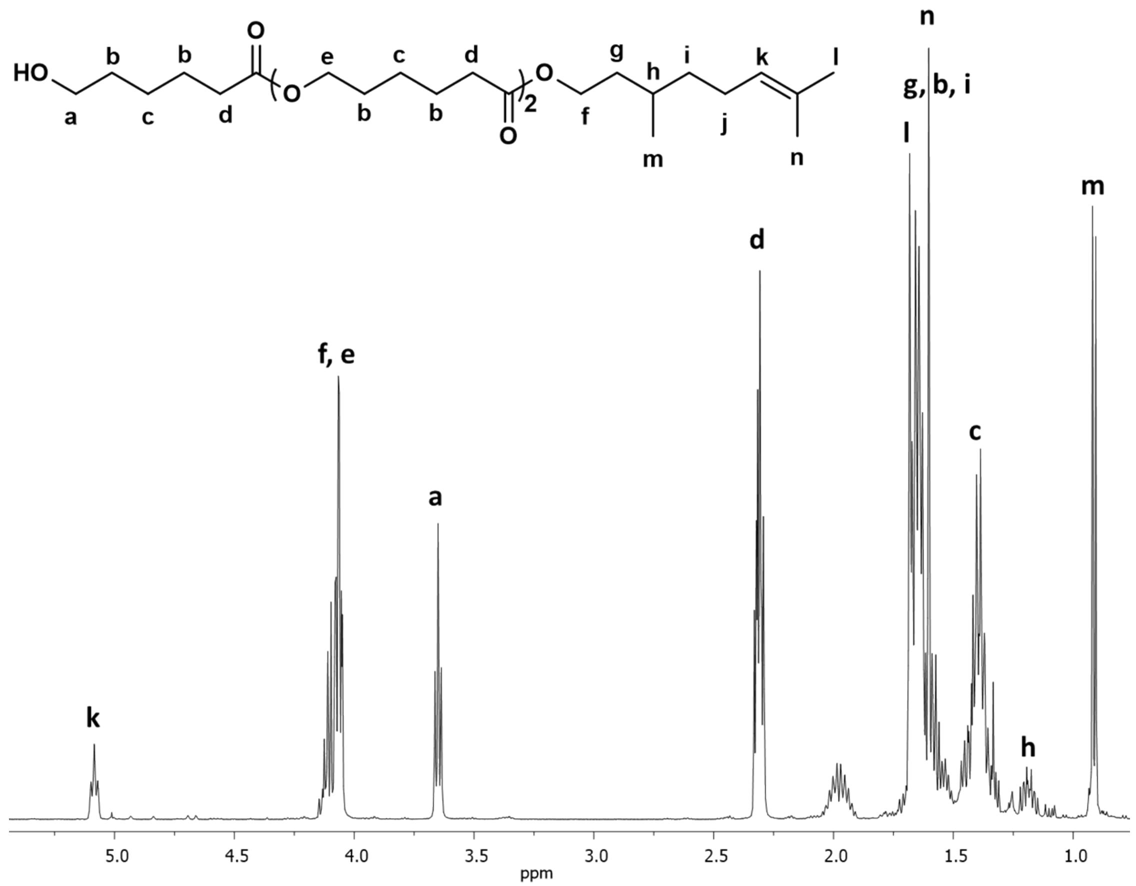

Fig. S10 ^{1}H NMR (500 MHz) spectrum of a trimer derived from *β*-citronellol as initiator $C_{10}C$-CL_3 (monodisperse specie, Table 2) in $CDCl_3$ isolated by FCC from $C_{10}C$-PCL (Table 1).

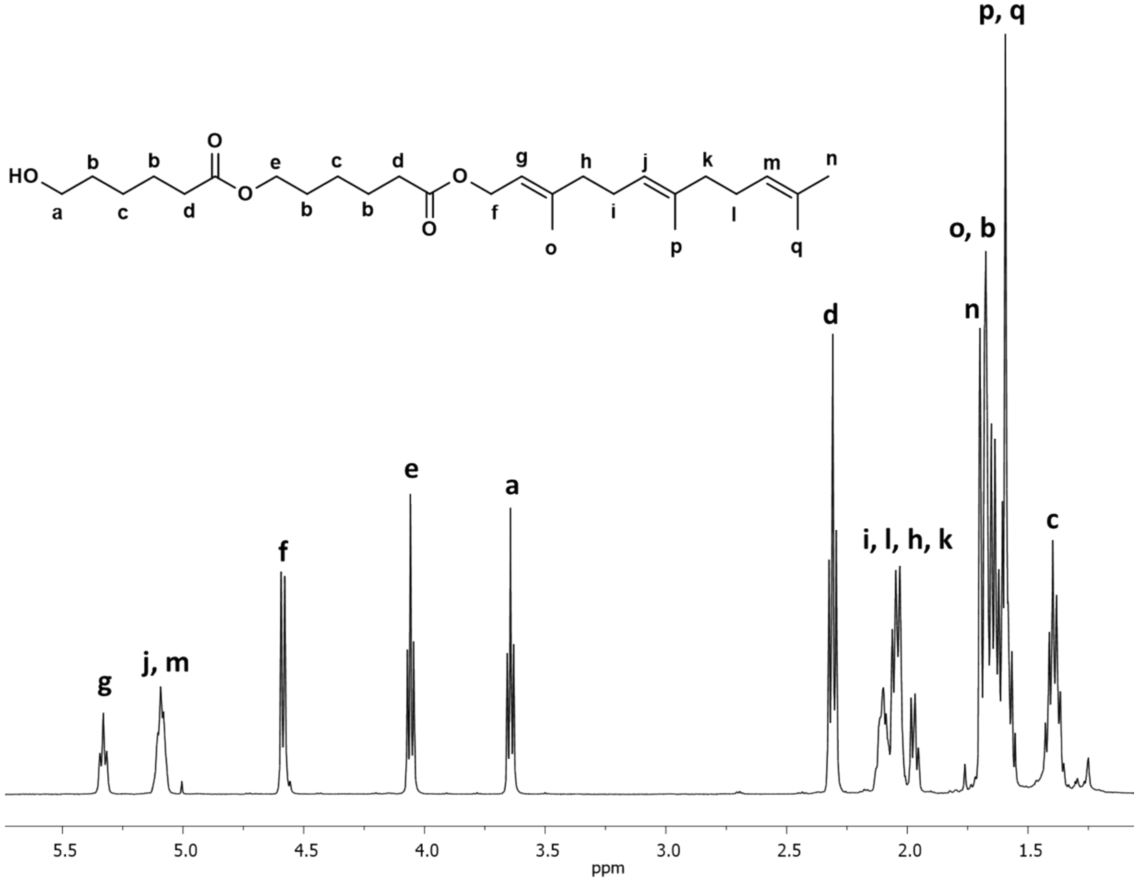

Fig. S11 ^1H NMR (500 MHz) spectrum of a dimer derived from farnesol as initiator $C_{15}F-CL_2$ (monodisperse specie, Table 2) in $CDCl_3$ isolated by FCC from $C_{15}F-PCL$ (Table 1).

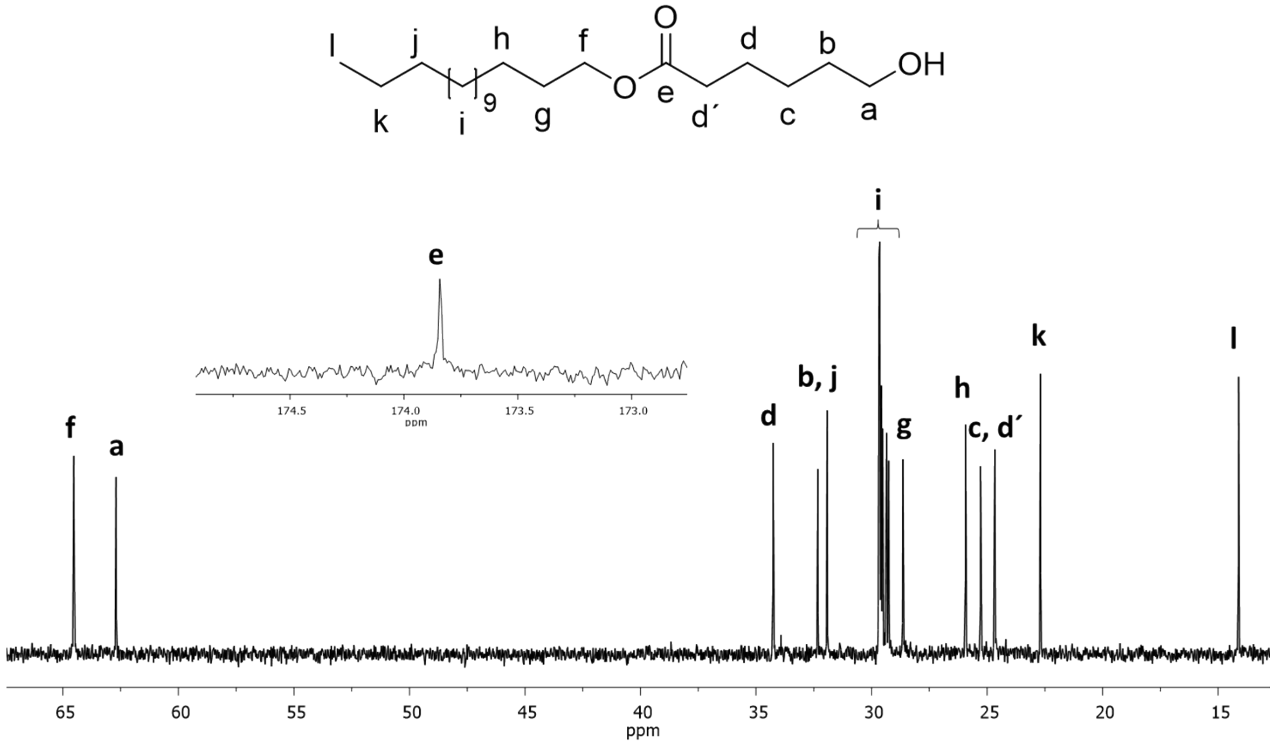

Fig. S12 ^{13}C NMR (500 MHz) spectrum of a monomer derived from 1-pentadecanol as initiator $C_{15}1P\text{-}CL_1$ (monodisperse specie, Table 2) in $CDCl_3$ isolated by FCC from $C_{15}1P\text{-}PCL$ (Table 1).

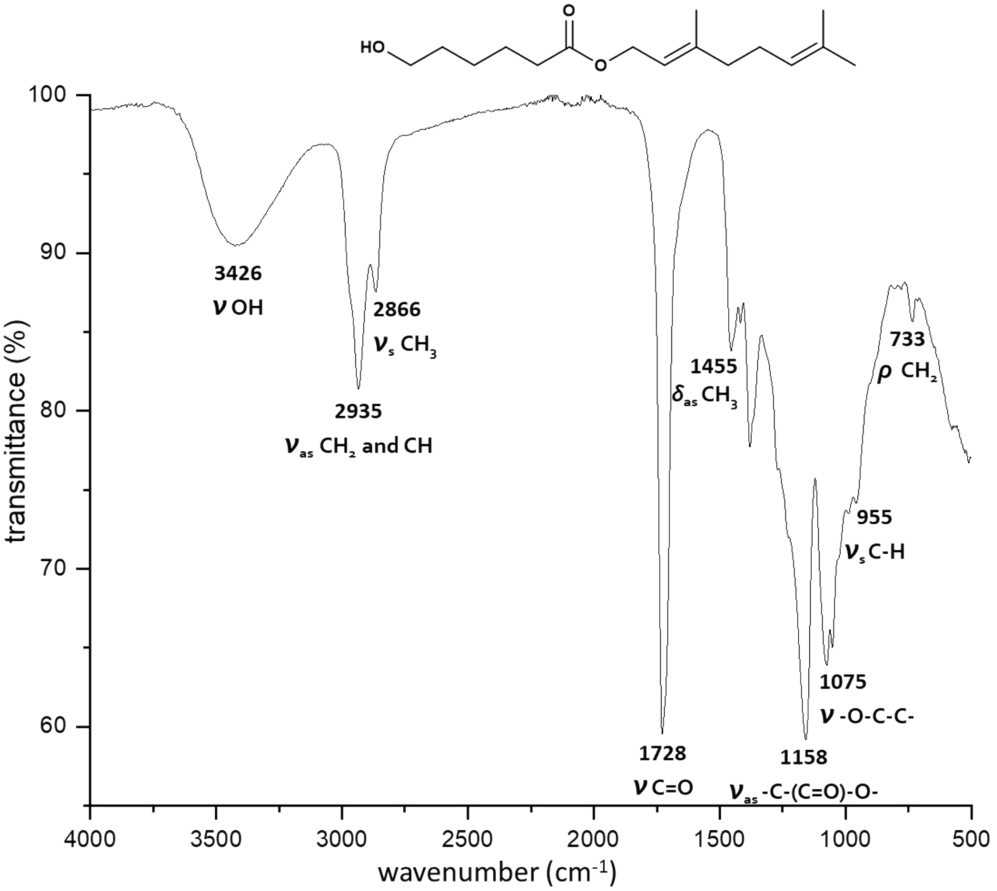

Fig. S13 FT-IR spectrum and assignment of bands from monodisperse monomer C_{10}G-CL_1.

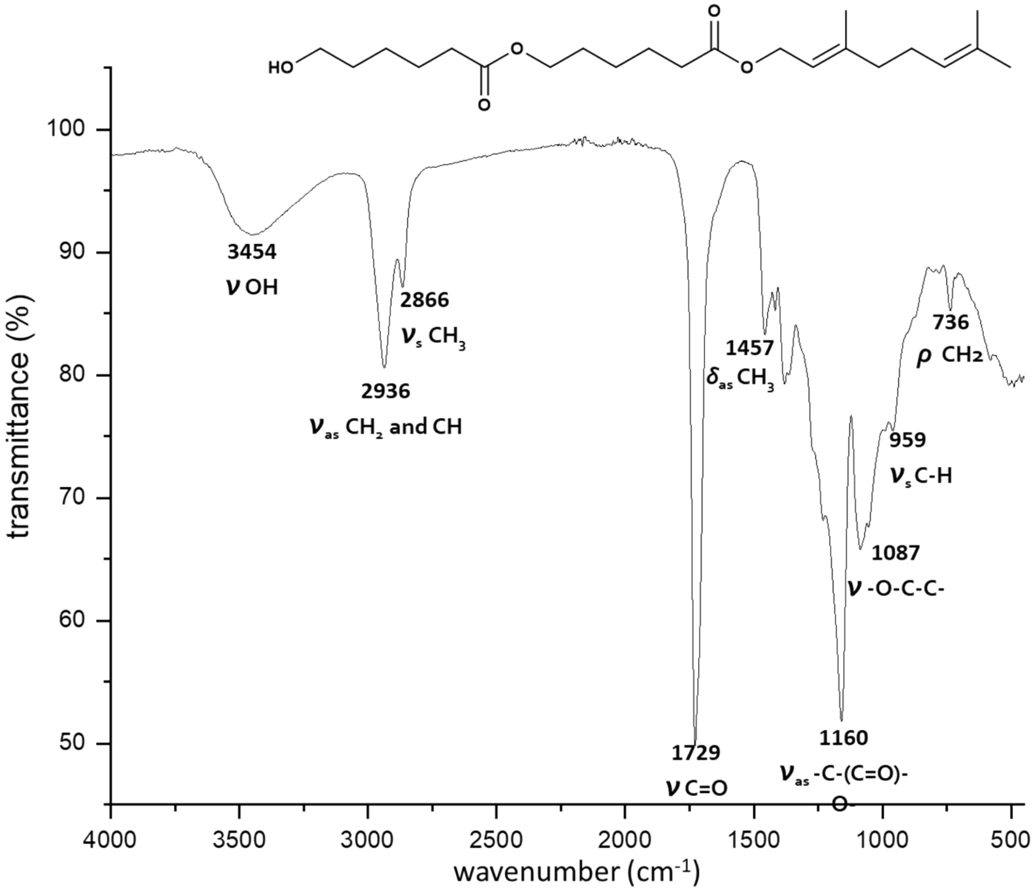

Fig. S14 FT-IR spectrum and assignment of bands from monodisperse dimer C_{10}G-CL_2.

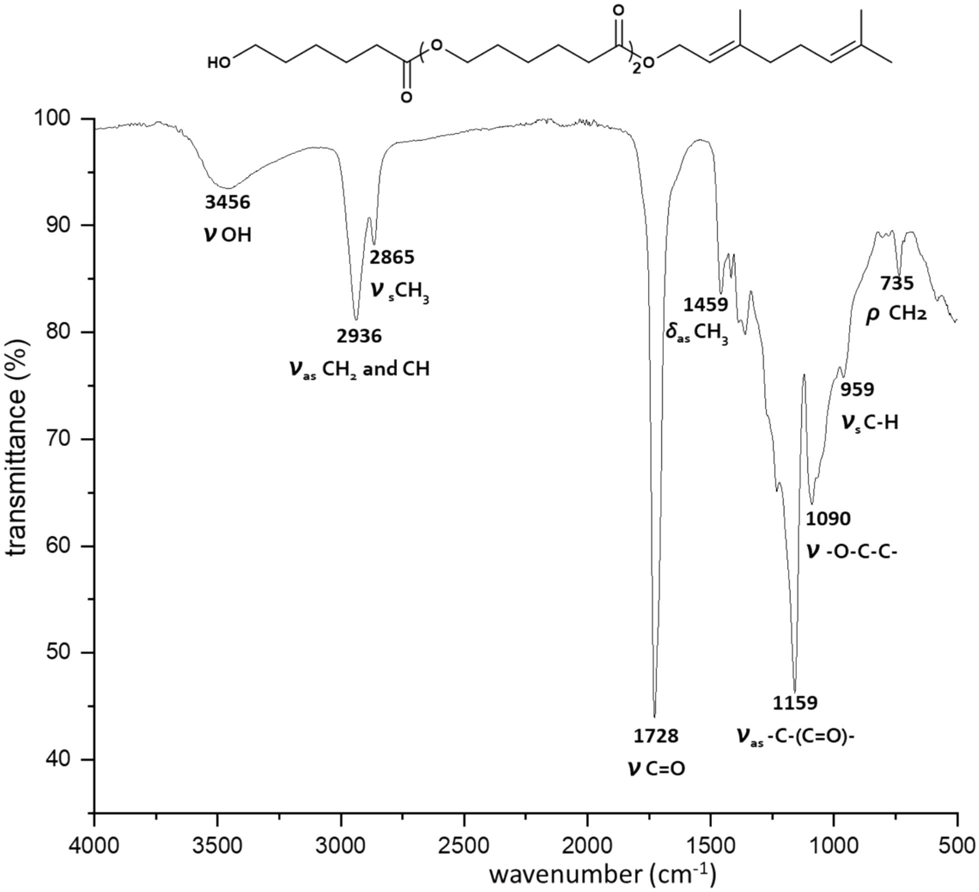

Fig. S15 FT-IR spectrum and assignment of bands from monodisperse trimer C_{10}G-CL_3.

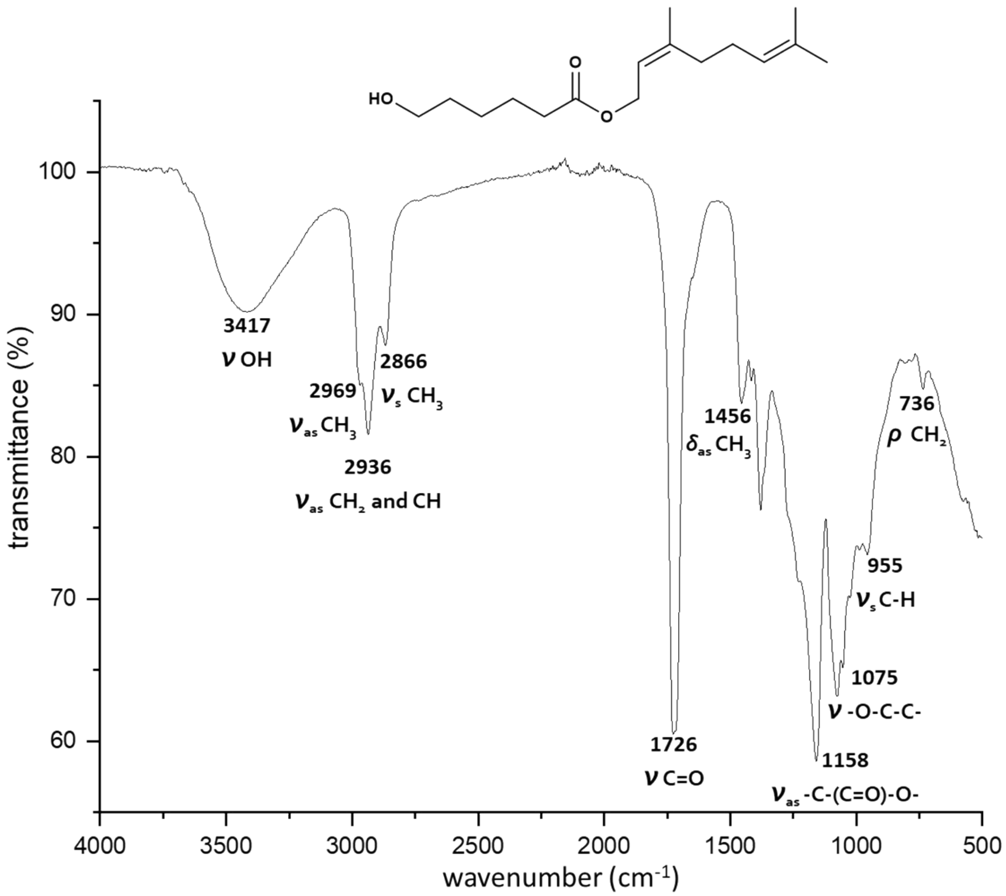

Fig. S16 FT-IR spectrum and assignment of bands from monodisperse monomer $C_{10}N\text{-}CL_1$.

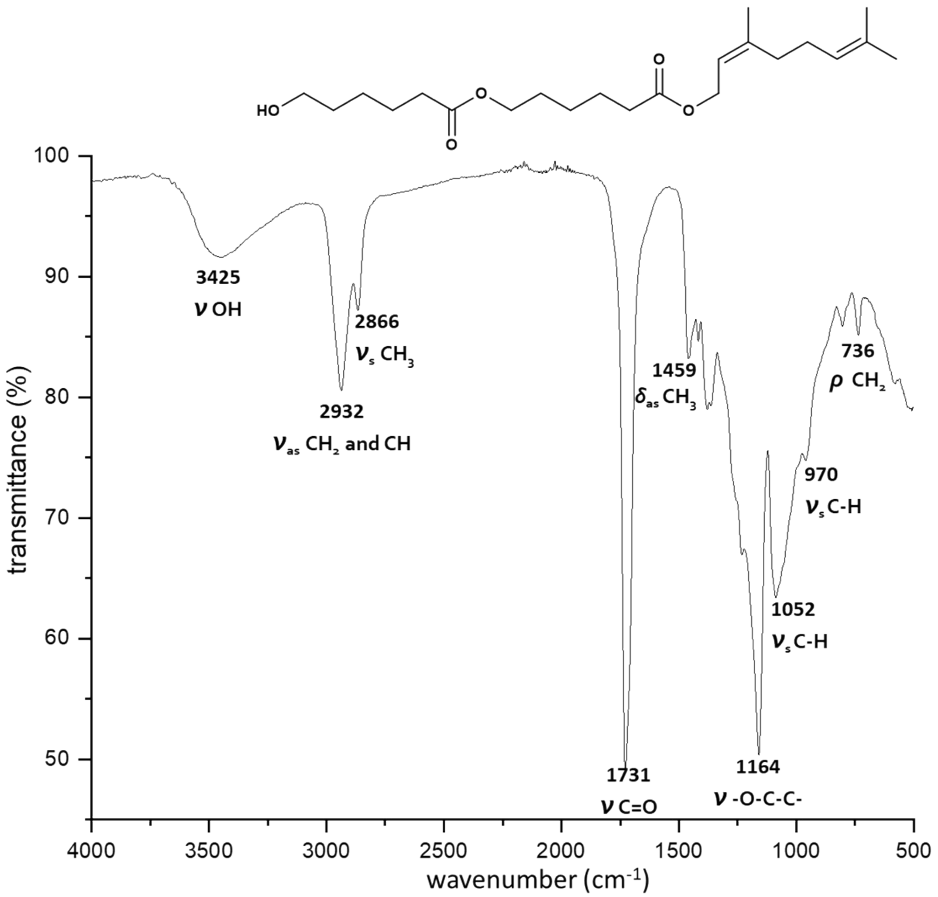

Fig. S17 FT-IR spectrum and assignment of bands from monodisperse dimer C_{10}N-CL_2.

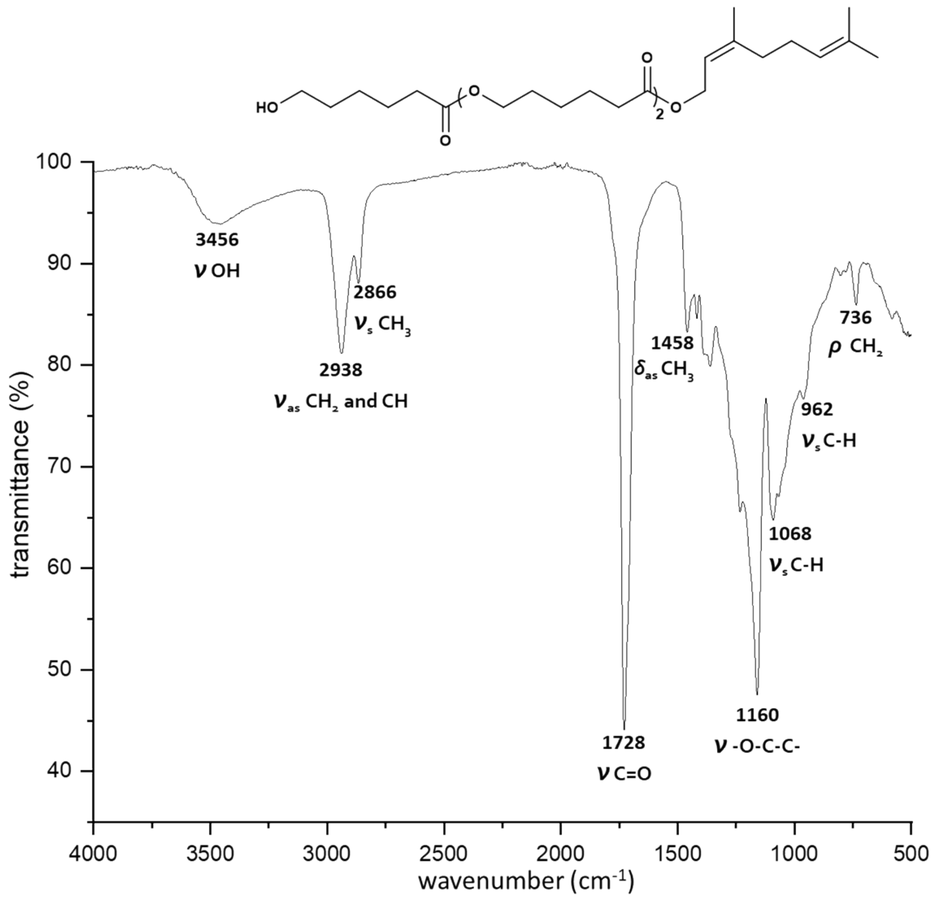

Fig. S18 FT-IR spectrum and assignment of bands from monodisperse trimer C_{10}N-CL_3.

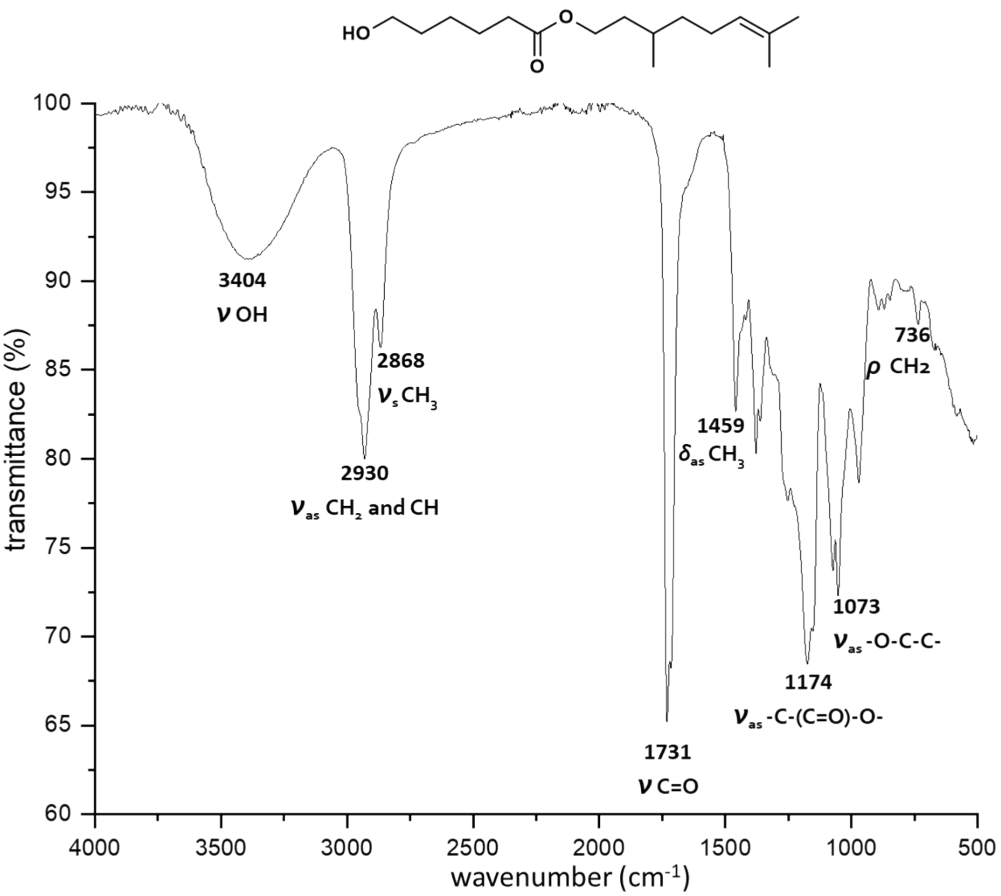

Fig. S19 FT-IR spectrum and assignment of bands from monodisperse monomer $C_{10}C\text{-}CL_1$.

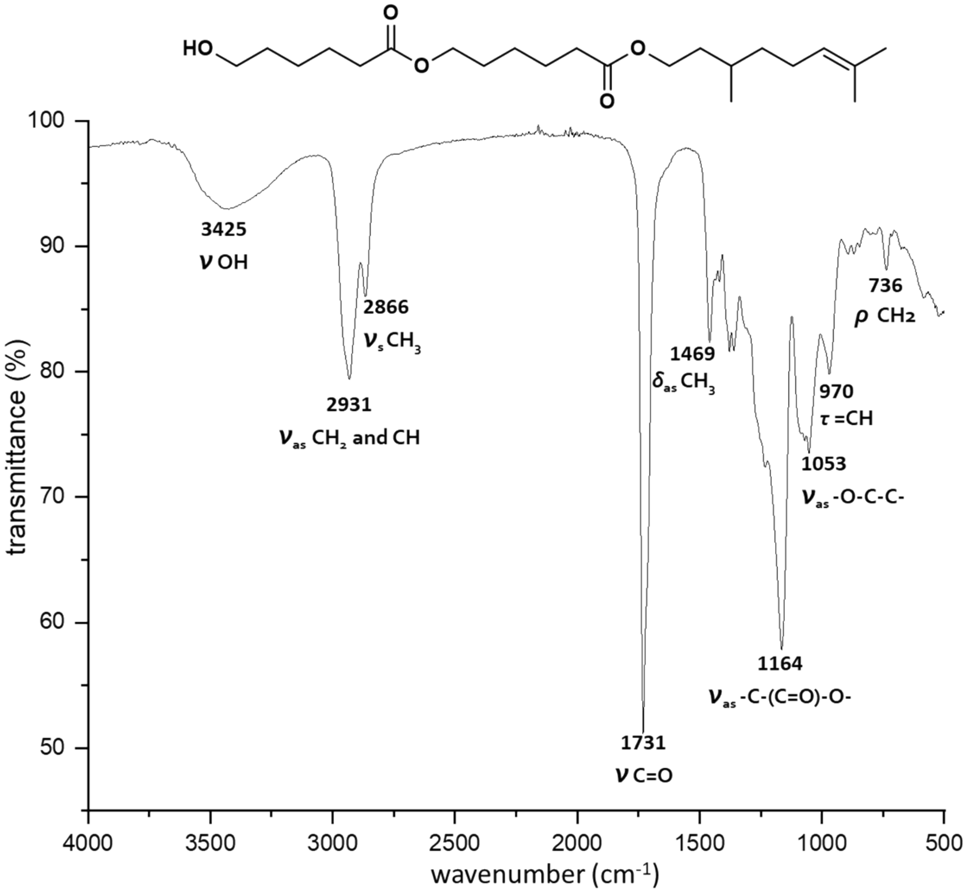

Fig. S20 FT-IR spectrum and assignment of bands from monodisperse dimer C_{10}C-CL_2.

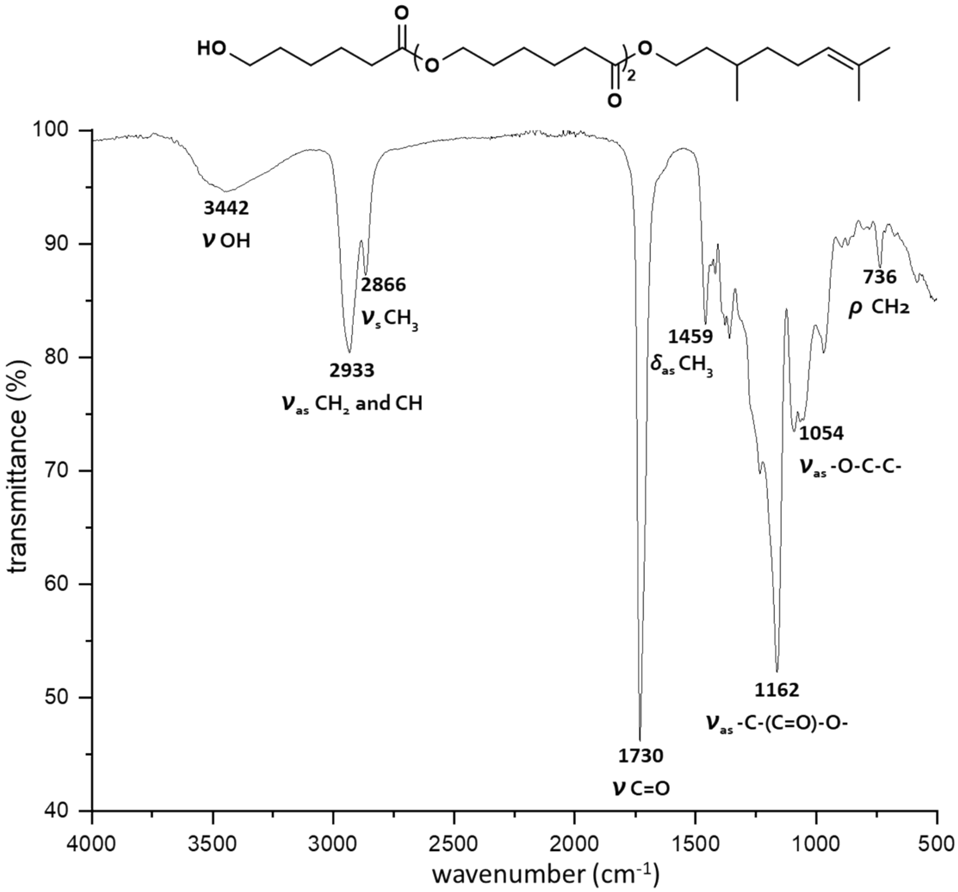

Fig. S21 FT-IR spectrum and assignment of bands from monodisperse trimer C_{10}C-CL_3.

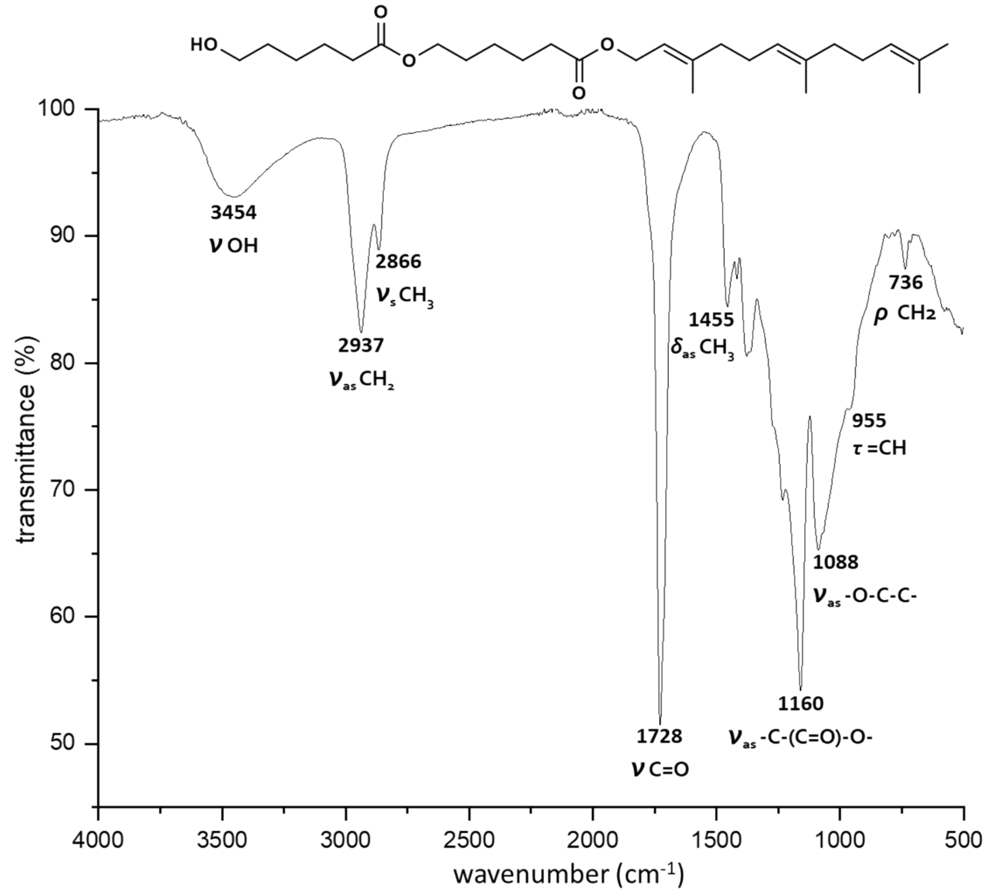

Fig. S22 FT-IR spectrum and assignment of bands from monodisperse dimer $C_{15}F$-CL_2.

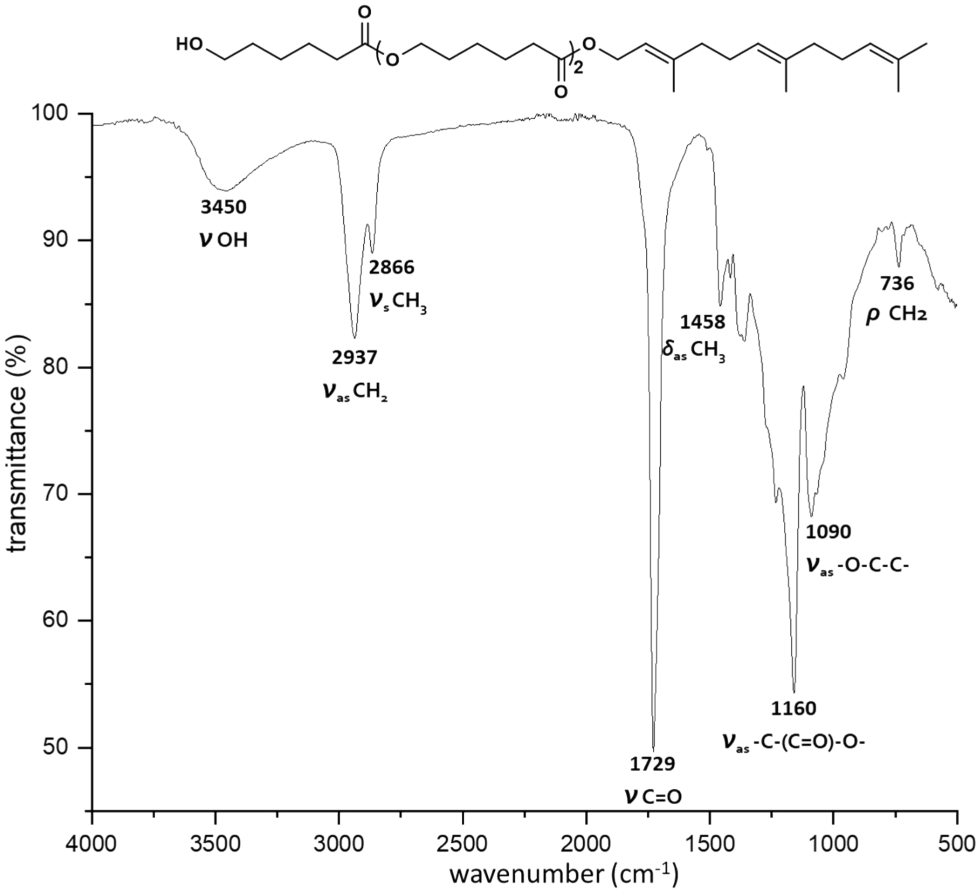

Fig. S23 FT-IR spectrum and assignment of bands from monodisperse trimer $C_{15}F$-CL_3.

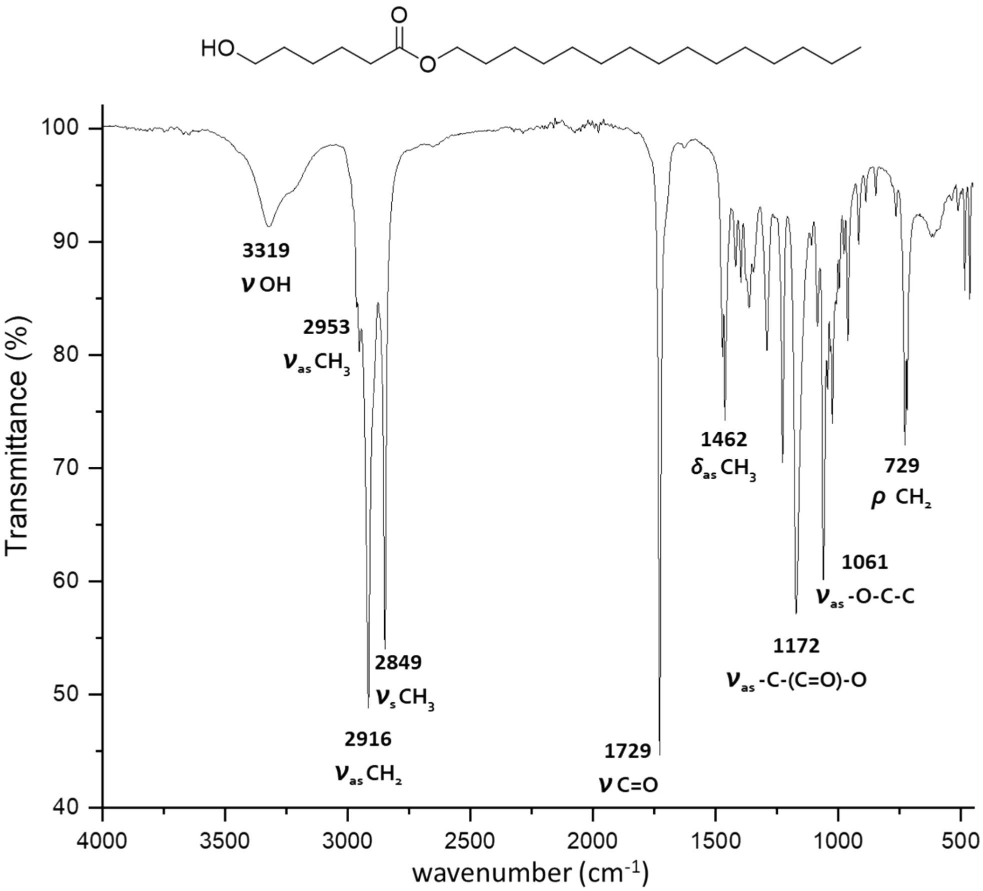

Fig. S24 FT-IR spectrum and assignment of bands from monodisperse monomer $C_{15}1P\text{-}CL_1$.

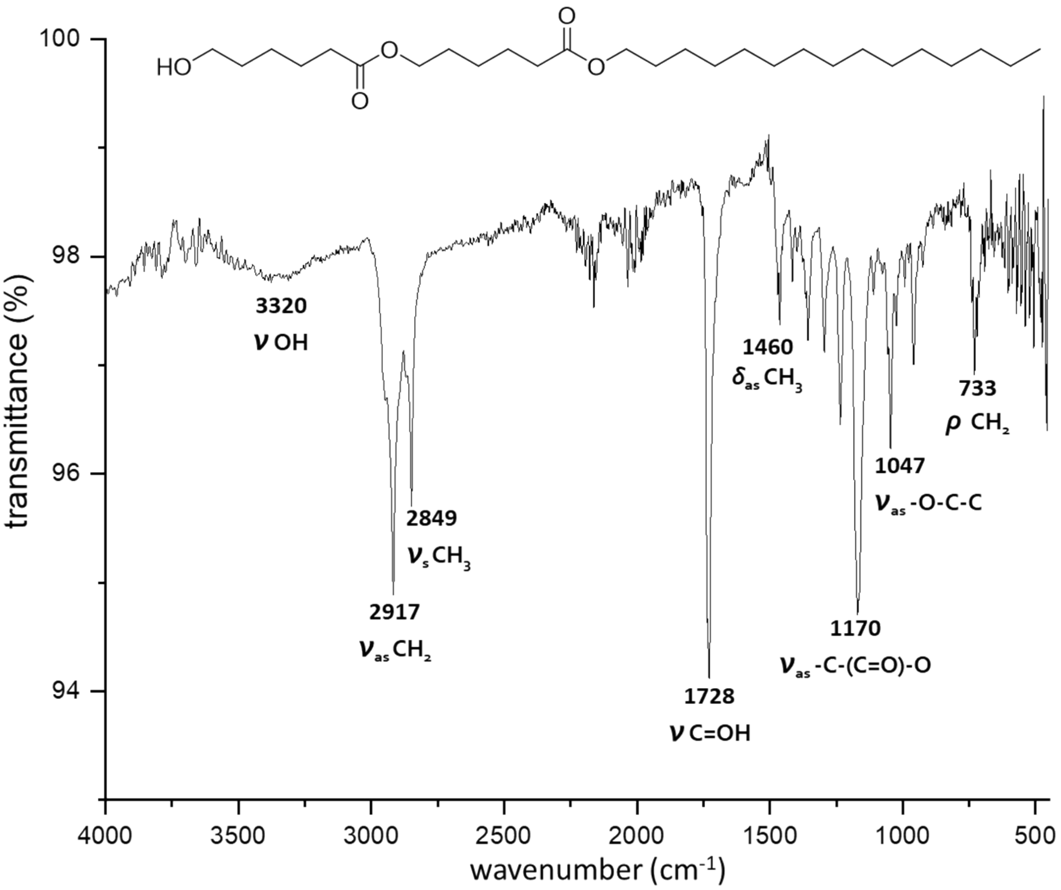

Fig. S25 FT-IR spectrum and assignment of bands from monodisperse dimer C_{15}1P-CL_2.

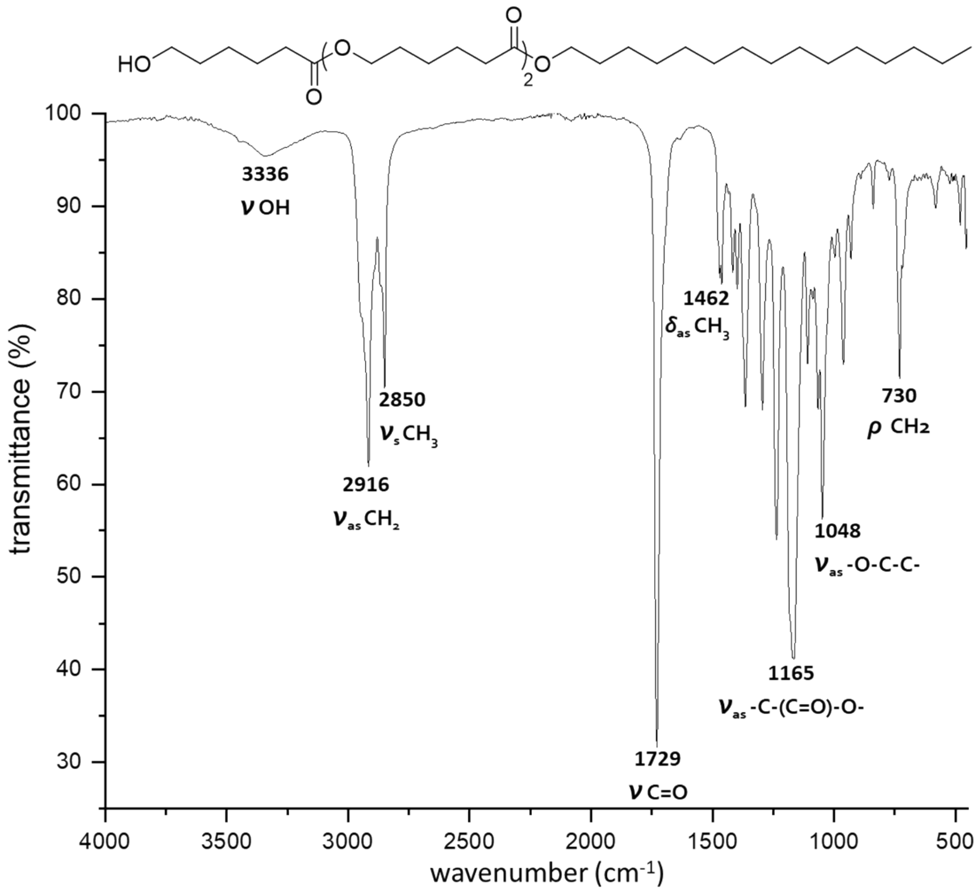

Fig. S26 FT-IR spectrum and assignment of bands from monodisperse trimer C_{15}1P-CL_3.

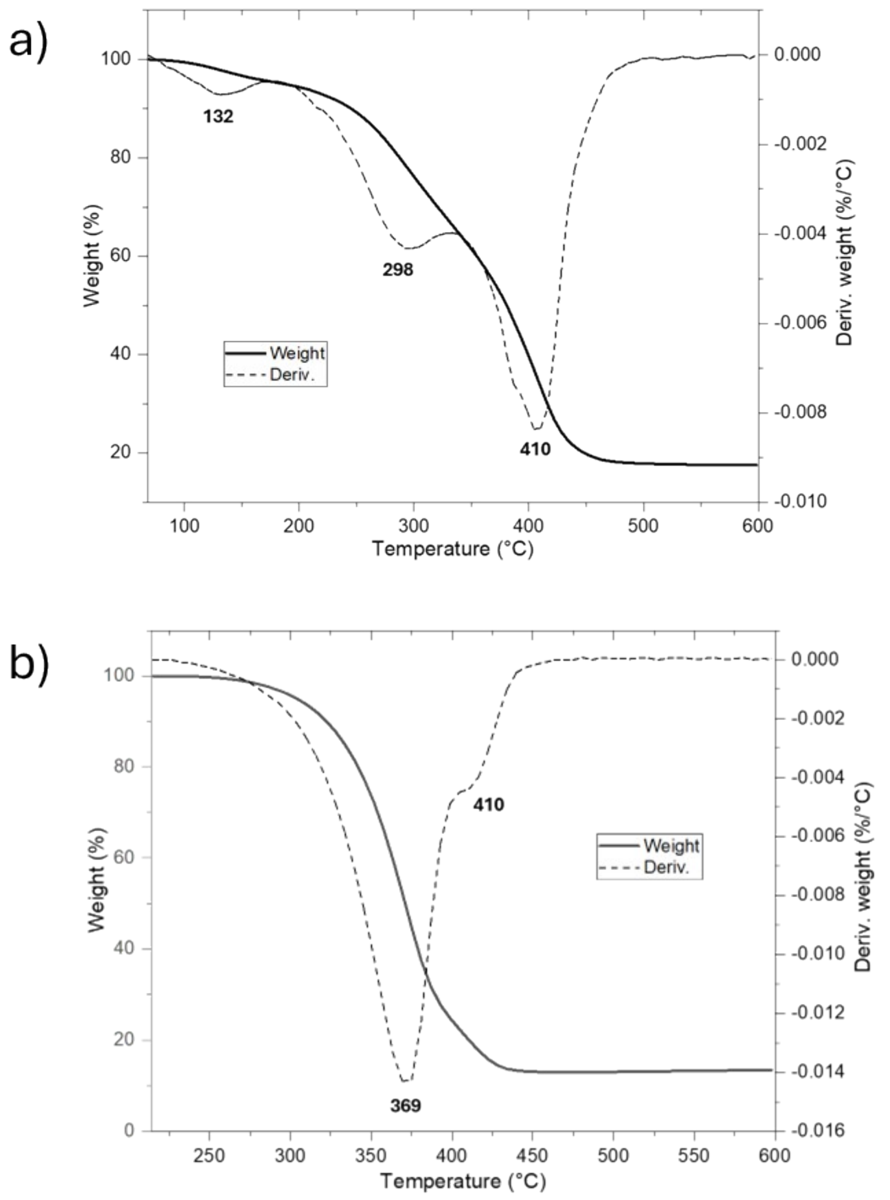

Fig. S27 Thermal degradation (TGA) of a) C_{15}F-CL_2 and b) C_{15}1P-CL_2.
